# Enhancing Phototoxicity in BODIPY‐Perylene Charge Transfer Dyads by Combined Iodination and Mesylation

**DOI:** 10.1002/chem.202403149

**Published:** 2024-11-09

**Authors:** Rhianne C. Curley, Ruben Arturo Arellano‐Reyes, James N. McPherson, Vickie McKee, Tia E. Keyes

**Affiliations:** ^1^ School of Chemical Sciences National Centre for Sensor Research Dublin City University Dublin Ireland; ^2^ Department of Physics Chemistry and Pharmacy University of Southern Denmark Campusvej 55 5230 Odense M Denmark

**Keywords:** BODIPY, Photodynamic therapy, Perylene, Spheroids, Iodination

## Abstract

The uptake and phototoxicity of a family of BODIPY‐perylene charge transfer dyads are compared in live cancer and non‐cancer cell lines to evaluate their performance in imaging and photodynamic therapy (PDT). The impact of iodination and mesylation of the meso position of the compounds on their optical properties, cell uptake and toxicity are compared. Notably, across all derivatives the probes were minimally dark toxic up to 50 μM, (the maximum concentration tested), but exhibited outstanding phototoxicity with nanomolar IC_50_ values and impressive phototoxic indices (PI, ratio of dark IC_50_ to light IC_50_), with best performance for the mesylated iodinated derivative MB2PI, which had a PI of >218 and >8.9 in MCF‐7 cells and tumour spheroids respectively. This is significantly higher than non‐iodinated analogue MB2P in MCF‐7 cells with an observed PI of >109 and slightly higher than MB2PI in spheroids with a PI of >8. This compound also showed interesting emission spectral variation with localisation that responded to stimulation of inflammation. Additional studies confirmed efficient singlet oxygen generation by the BODIPYs, suggesting a Type II mechanism of phototoxicity. Overall, the data indicates that combining charge transfer and iodination is an effective strategy for enhancing phototherapeutic capacity of BODIPY PS.

## Graphical abstract

We report a family of BODIPY‐perylene charge transfer compounds with and without iodination and/or a mesityl group. The BODIPY derivatives reported show high phototoxicity in both 2D cell cultures and multicellular tumour spheroids, where MB2PI (iodinated BODIPY‐perylene with a mesityl group) was the most phototoxic derivative, indicating that combining strategies to enhance phototoxicity can yield positive results.

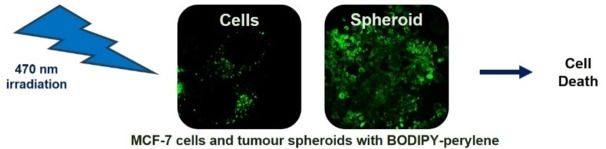

## Introduction

BODIPY (boron‐dipyrromethene) derivates are versatile luminophores^[1]^ that have been extensively studied as candidates for bio‐imaging^[2]^ and intracellular sensing.[^3^, ^4^, ^5^, ^6^, ^7^] This extensive interest has been driven by their attractive optical properties, including high absorption coefficients and emission quantum yields at wavelengths that can be tuned from visible to NIR through selective synthetic modification of the BODIPY core.[^8^, ^9^, ^10^, ^11^, ^12^] The high emission quantum yields of many BODIPY derivatives is associated with their low quantum yield of inter‐system crossing (ISC) which also limits their phototoxicity through singlet oxygen (^1^O_2_) pathways.^[13]^ There is nonetheless, growing interest in BODIPYs as metal‐free photosensitisers (PSs) for photodynamic therapy (PDT)[^14^, ^15^, ^16^] with several strategies available to promote their formation of triplet states. These include through substitution with heavy atoms and implementation of charge transfer in the compound. In BODIPY derivatives, such strategies have expanded their applications in PDT,^[17]^ and triplet‐triplet annihilation up‐conversion (TTA‐UC).[^18^, ^19^, ^20^] In ISC promoted through spin‐orbit charge transfer intersystem crossing (SOCT‐ISC), introducing donor‐acceptor moieties to the BODIPY core enables photoinduced electron transfer between the subunits, forming a singlet charge‐transfer state that undergoes charge recombination, thereby facilitating the transition to a triplet excited state.^[21]^ This approach is attractive in BODIPY derivatives as they can behave as either excited state donor or acceptor as demonstrated across numerous donor‐acceptor structures.[^22^, ^23^] Furthermore, optically, charge transfer transitions build useful spectroscopic properties into the derivatives, including large Stokes shifts and environmental sensitivity. We and others have recently developed BODIPY‐perylene charge transfer compounds capable of SOCT‐ISC that is promoted by the orthogonality of the donor perylene and acceptor BODIPY.[^24^, ^25^] We further demonstrated that SOCT‐ISC can be used to facilitate TTA‐UC and ISC promoted by co‐substitution with iodine via the heavy atom effect.^[26]^ Additionally, iodination has been applied to promote ISC and ^1^O_2_ generation by BODIPY derivatives to promote phototoxicity.[^27^, ^28^] Nagano et al. were amongst the first to report an iodinated BODIPY for PDT in 2005,^[29]^ since then many groups have studied these systems for PDT including Liu et al. who prepared an iodinated BODIPY targeted to lysosomes for PDT with a reported IC_50_ of 2.34 μM (525 nm, 4 mW/cm^2^, 15 min).^[30]^ We have shown that SOCT can combine with heavy atom effect to promote ISC, which in principle can also increase phototoxicity through ^1^O_2_ generation.^[26]^

**Figure 1:**
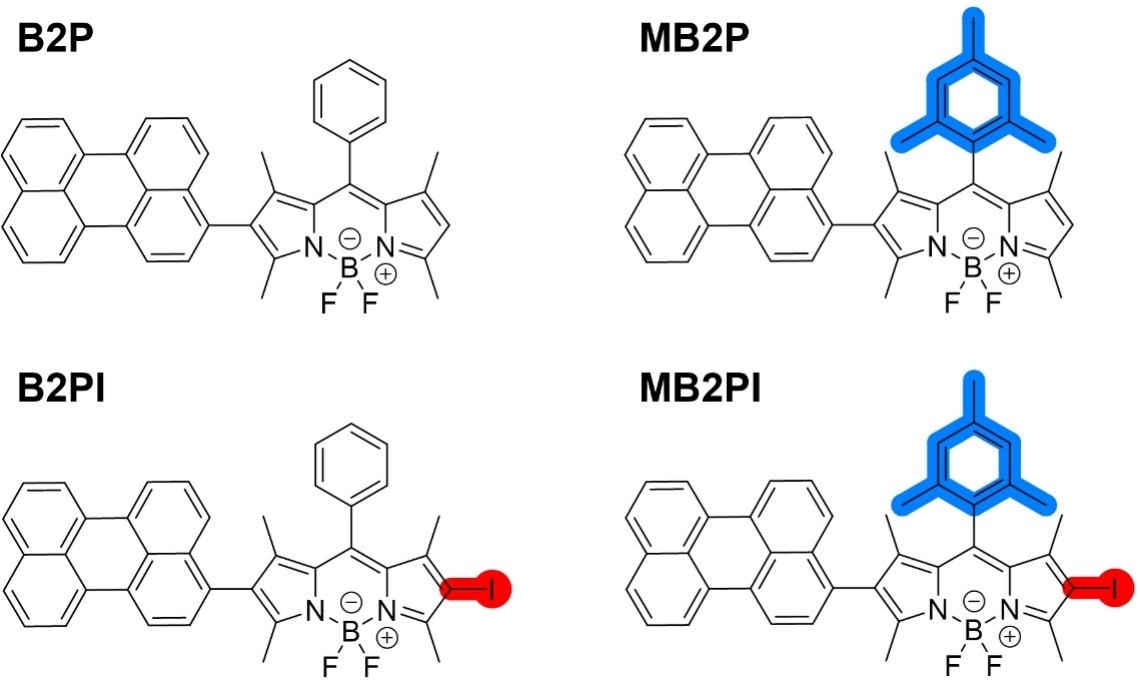
Chemical structures of B2P, B2PI, MB2P and MB2PI studied in this work.

Wang and co‐workers demonstrated that a BODIPY‐perylene derivative, BDP‐pery‐2 (referred to as B2P herein), exhibited high phototoxicity towards HeLa cells under an irradiation dose of 9.6 J/cm^2^ (500 nm irradiation) with a phototoxic index (PI) of 1041.^[24]^ Thus, in extending the family of BODIPY‐perylene derivatives, we were interested to investigate the phototoxic capacity of B2P and an associated family of BODIPY‐perylene PSs developed by our group (Figure 1) with different extents of substitution against another cancer cell line, comparing iodine substituted charge transfer derivatives to their noniodinated counterparts.

Methylation of BODIPY at the meso phenyl group can enhance the emission quantum yield by reducing non‐radiative decay rates, potentially increasing molecular brightness for imaging.^[31]^ Methyl substitution can also increase the lipophilicity and improve membrane permeability, facilitating enhanced cellular uptake of the PS resulting in higher intracellular concentrations and can promote localisation to membrane or lipid droplets.^[32]^ For example, in methyl‐aminolevulinic acid (MAL), a lipophilic derivative of the clinically approved PS 5‐aminolevulinic acid (ALA), methylation led to enhanced membrane permeability and overall therapeutic efficacy.^[33]^ 1,3,5‐trimethylbenzene or meso‐mesityl substituted BODIPYs, may offer comparable benefits. In the present case, the mesityl group was also introduced to decrease the non‐radiative relaxation pathways and improve charge transfer character of the excited state. We compare the phototoxic effects of the iodinated BODIPY compounds B2PI with MB2PI and their noniodinated analogues in MCF‐7 cells, a commonly studied breast cancer cell line, and CHO (Chinese Hamster Ovary) non‐cancer cells.

While BODIPY‐derivatives have been widely explored as PSs in PDT, their *in vitro* evaluation has mainly focused on 2D cell culture. 3D spheroid models provide a much closer mimic of the tumour environment for PS including penetration, distribution, and phototoxic effects, but are much less commonly employed.^[34]^ Campbell et al. investigated a tellurophene‐appended BODIPY, which showed high phototoxicity in HeLa spheroids (525 nm, 23.60 mW/cm^2^, 5 min),^[35]^ Ballestri and Marras et al. tested the phototoxicity of their free and poly‐methyl‐methacrylate‐bounded BODIPYs in HCT116 colon cancer and MCF‐7 spheroids^[36]^ and Schneider et al. grew HeLa spheroids to evaluate their BODIPY‐based PSs which showed promise as agents for photothermal and photodynamic therapies.^[37]^

In this contribution, we report on the synthesis and spectroscopy of mesylated BODIPY‐perylene derivatives extending the family of BODIPY‐perylene derivatives with and without iodination. Strategies using charge transfer and iodination have been exploited to enhance phototoxicity of BODIPY sensitisers, but there has been limited exploration in combining both approaches to amplify phototoxic effects. Thus, we examine and compare uptake, distribution, dark and phototoxicity of the derivates in MCF‐7 (dsmz, ACC‐155) and CHO cells with the associated compounds without substitution. The phototoxic potential of the family of probes was assessed under normoxic and hypoxic (MB2PI) conditions, and phototoxic index (PI) values determined. The mechanism of phototoxicity was determined using ^1^O_2_ scavenger DPBF in solution and ROS dye H_2_DCFDA *in cellulo*. Cellular uptake of the BODIPY‐perylene derivatives was further confirmed in MCF‐7 spheroids by confocal imaging and toxicity was assessed using plate‐based assays.

## Results and Discussion

### Synthesis and Photophysical Characterisation

**Scheme 1:**
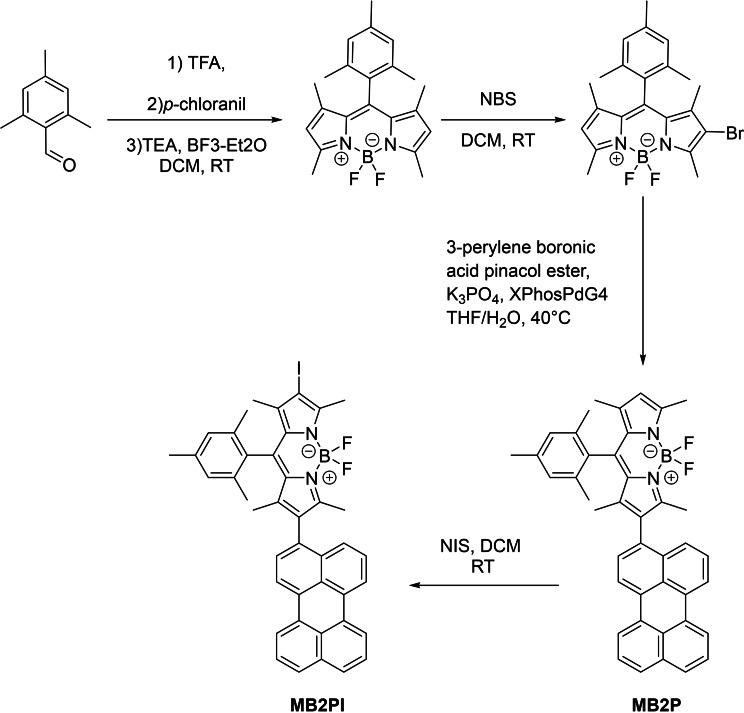
Synthetic strategy to prepare MB2P and MB2PI.

The family of BODIPY‐perylene derivates studied, B2P, B2PI, MB2P and MB2PI, are shown in Figure 1. MB2P and MB2PI were prepared according to Scheme 1, while the synthesis and photophysical data for B2P and B2PI were reported recently.[^25^, ^26^]

### Crystallography

**Figure 2:**
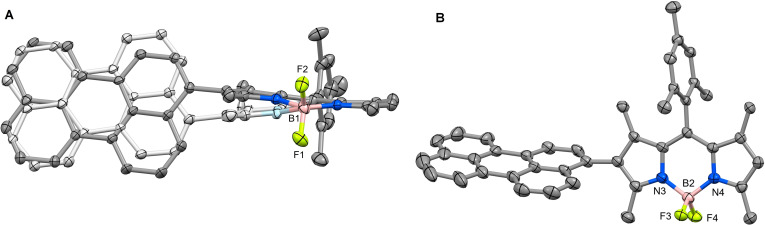
(A) MB2P molecule 1, showing disordered perylene ring (minor component shown with paler colours) and (B) MB2P molecule 2. Hydrogen atoms omitted for clarity, 30 % probability ellipsoids.

Crystallography can offer valuable insight into the relative orientation of donor and acceptor moieties in SOCT‐ISC. Crystals of MB2P⋅CHCl_3_ were obtained for crystallography by vapour diffusion. The asymmetric unit (Figure S11) contains two independent C_42_H_35_BF_2_N_2_ molecules and some disordered chloroform solvate. Figure 2A shows molecule 1, in which the perylene group is disordered by rotation of 180° about the C−C bond linking it to the BODIPY section, and the site occupancies are refined to 0.74 and 0.26 for the major and minor components, respectively. A slight displacement of the linked 5‐membered ring allows a close spatial overlap of the two perylene components. The second molecule (Figure 2B) is not significantly disordered. The conformations of the two molecules are similar and the small differences (Figure S15) can probably be ascribed to intermolecular interactions in the solid state. In each case the mean plane of the perylene group is rotated with respect to the mean plane of the BODIPY core, these interplanar angles are 69.8(1)° and 73.5(3)° for the major and minor components of molecule1 and 73.23(6)° for molecule 2. These values are similar to the equivalent interplanar angles for the two independent molecules in the structure of B2P (73.54(3)° and 74.51(4)°).^[26]^ The lower value for the major component of molecule 1 may be a consequence of π‐π stacking (Figure S16).

### Absorption and Emission Properties

**Figure 3:**
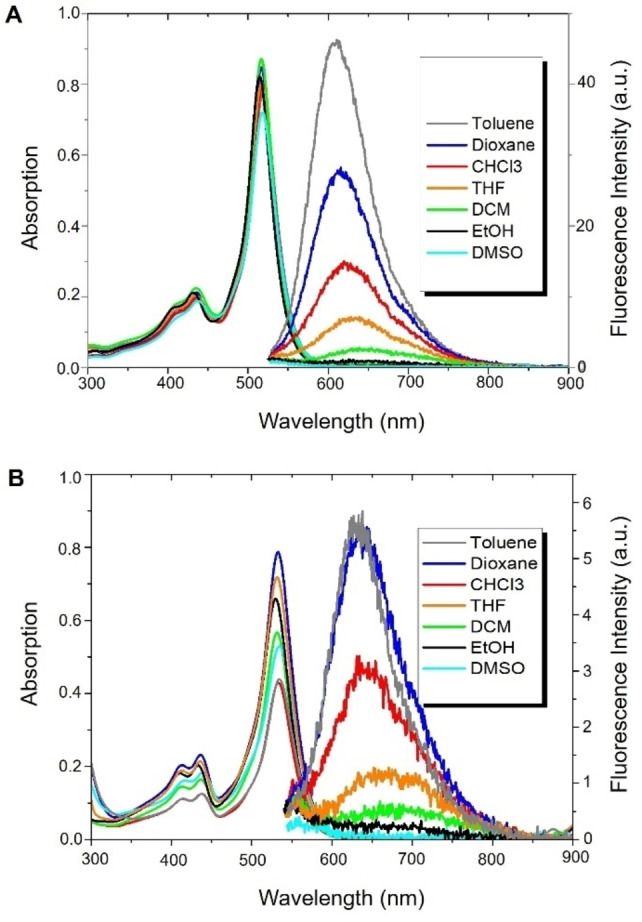
Right, Absorption spectra of (A) MB2P and (B) MB2PI in different solvents at 10 μM, and, left, their respective emission. Excitation wavelength 532 nm, data recorded with 2.5–2.5 nm slit widths.

The absorption and emission spectra of MB2P and MB2PI are shown in Figure 3, in a range of solvents across polarities. The optical and photophysical data for the complete family of compounds in toluene and dioxane are summarised in Table 1. The absorption maxima are centred at approximately 515 nm (MB2P) and 532 nm (MB2PI) in dioxane and are very similar to those of B2P and B2PI, reported previously to exhibit maxima at 515 nm and 531 nm in dioxane. The absorption maxima is largely unaffected by solvent, similar to B2P/B2PI. The extinction coefficient is also modestly affected for MB2P but does vary with solvent for MB2PI, although the change is not correlated with solvent polarity. All compounds also show characteristic perylene absorption features between 400 and 430 nm. Also consistent with this previous study, the emission spectra are strongly solvent dependent. Intensely emissive from non‐polar media, MB2P and MB2PI are minimally emissive in polar (ethanol and DMSO) and aqueous media, (with 10 % DMSO for solubilisation). For example, under matched absorption conditions used to generate Figure 3, the emission intensity from water was 0.13 % (MB2P) and 1.6 % (MB2PI) of the emission intensity from toluene (see Figure S20).

**Table 1:** Photophysical properties of B2P, B2PI, MB2P and MB2PI in 1,4‐dioxane and toluene.

	Solvent	λ_abs_ (nm)	λ_em_ (nm)	Stokes Shift (cm^−1^)	ϵ (M^−1^ cm^−1^)	*Φ_f_* ^[b]^	Mol. Bright	τ_1_ (ns)	Amp (%)	τ_2_ (ns)	Amp (%)	τ_ave_^*^ (ns)	k_r_^[c]^ (10^7^ s^−1^)	k_nr_^[c]^ (10^7^ s^−1^)
**B2P [a]**	Toluene	518	616	3071	80400	0.21	16884	2.5±0.0		—		2.5±0.01	2.6	79
	Dioxane	515	625	3417	73200	0.09	6588	4.1±0.2	5.3	1.8±0.0	94.7	2.0±0.0	4.6	44
**B2PI [a]**	Toluene	534	636	3003	63300	0.03	1899	2.9±0.1	7.2	0.7±0.01	92.8	1.2±0.0	8.5	32
	Dioxane	531	641	3232	67600	0.03	2028	3.8±0.1	94.6	0.6±0.01	5.4	1.4±0.01	1.8	68
**MB2P**	Toluene	517	612	3002	89300	0.36	32148	2.8±0.0	–	–	–	2.8±0.0	12.9	23
	Dioxane	515	614	3131	89200	0.23	20516	3.1±0.1	–	–	–	3.1±0.1	7.4	25
**MB2PI**	Toluene	534	630	2854	72600	0.09	6534	1.6±0.1	5.6	0.7±0.01	94.4	0.8±0.0	11.2	114
	Dioxane	532	635	3049	72900	0.06	4374	4.0±0.1	2.5	0.7±0.01	97.5	1.1±0.0	5.73	85

^[a]^ B2P and B2PI data previously published^[26]^ ^[b]^ Absolute quantum yields were obtained in an integrating sphere and have a±2 % error ^[c]^ Radiative decay rates calculated using kr=ΦF/τave. Non radiative decay estimated from knr=(1‐Φ)* Intensity weighted average lifetime. Amp=Amplitude.

Based on both DFT calculations reported for B2P/B2PI, and the observation of perylene‐BODIPY cation‐anion radical pair in femtosecond spectroscopy of these compounds, the absorbance and emission are similarly ascribed to perylene‐to‐BODIPY charge transfer transitions, which accounts for the solvent dependent emission behaviour.[^26^, ^38^] The compounds also all show large emission Stokes shifts, of approximately 3000 cm^−1^. While behaviour is very similar across B2P/B2PI and MB2P/MB2PI, the latter show modest blue shifts in their emission maxima, and MB2P/MB2PI show significant increases in quantum yields compared to B2P/B2PI. Correspondingly, as shown in Table 1 the molecular brightness, an important parameter in imaging probes, is dramatically improved by methylation of the meso phenyl group and the impact is greatest for the iodinated compounds where mesitylation results in close to 30x increase in molecular brightness for MB2PI compared to B2PI in both toluene and dioxane. The affects are likely attributed to reduced rotational degrees of freedom due to methyl substitution of the meso phenyl group and this is reflected in reduced k_nr_ for MBP2 in Table 1, but not MB2PI where k_nr_ increases are likely reflective of increased triplet population.

Fluorescence lifetimes are within expected ranges for BODIPY derivatives and are comparable between B2P and MB2P. Notably, as previously reported for B2PI, MB2PI exhibits biexponential fluorescence decays in both dioxane and toluene, whereas MB2P shows monoexponential decay in both media. Deaeration, within experimental error, exerts no influence on the emission lifetimes of the compounds. The short‐lived component of the decay at 0.7 ns, dominates for MB2PI in both solvents and while origin of this component is unclear, similar behaviour was noted in B2P. Therein, it was suggested that different rotamers may be at play. In the case of the MB2P derivatives the rotational degrees of freedom are expected to be confined to the perylene moiety as the methylated phenyl group at the meso position is expected to be rotationally locked.[^39^, ^40^] Therefore, it is likely, as reported for B2P/B2PI, that the complexity of response for the iodinated species may arise from solvent influence on population and lifetime of the triplet state.^[25]^

### Cellular Uptake and Localisation

**Figure 4:**
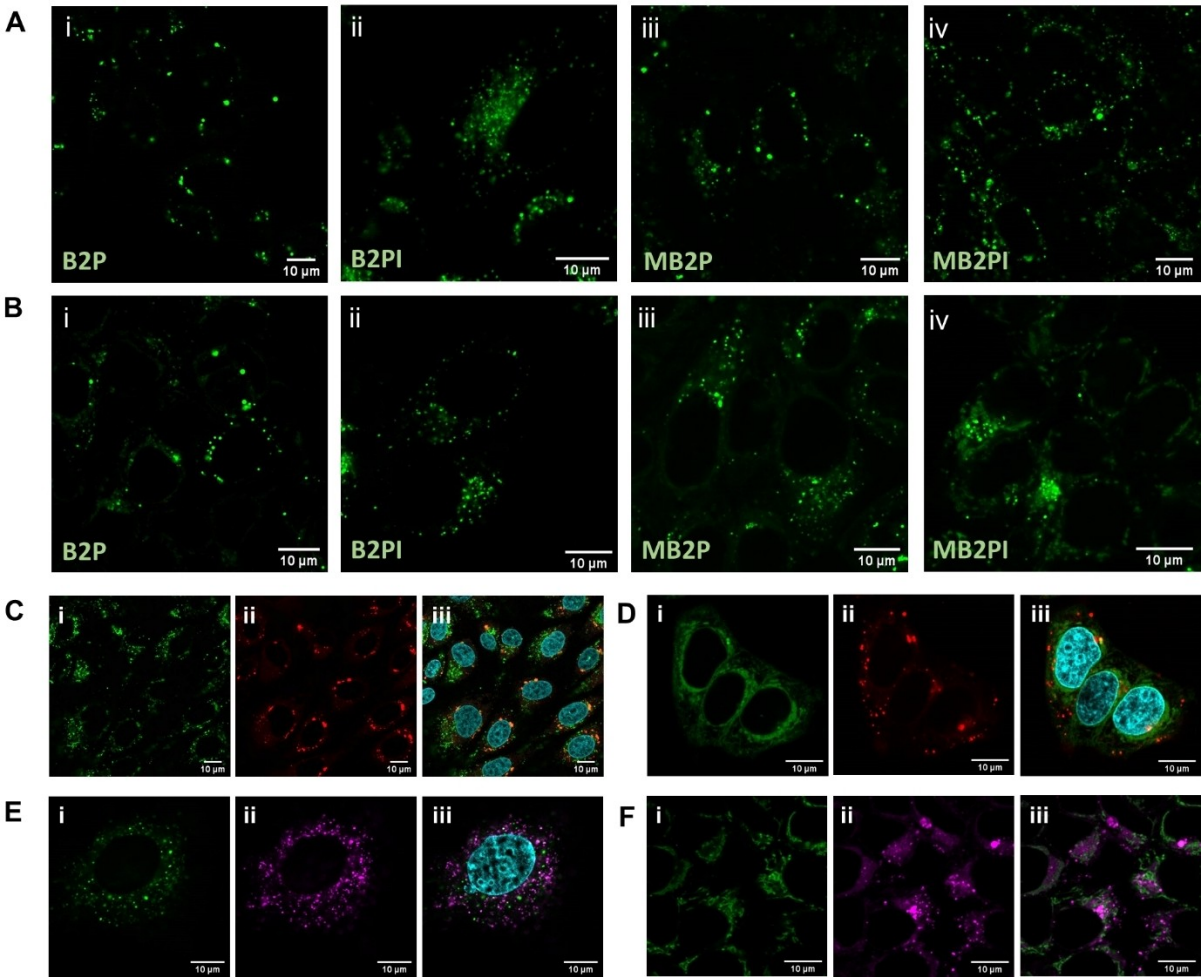
Confocal images of (A) CHO and (B) MCF‐7 cells with 10 μM (i) B2P and (ii) B2PI and 30 μM (iii) MB2P and (iv) MB2PI after a 17 h incubation at 37 °C. The λmax of each BODIPY was selected for excitation and emission was collected between 550–700 nm. Co‐localisation imaging of BODIPY 493/503 with (C) MB2P and (D) MB2PI and LysoTracker Deep Red with (E) MB2P and (F) MB2PI are shown where BODIPY 493/405 is indicated in (ii) imaging in red and LysoTracker Deep red is shown in (ii) in magenta. Hoechst 33342 indicates the nucleus in C, D and E (iii).

The intracellular uptake of the BODIPY derivatives was assessed in two mammalian cell lines, CHO (non‐cancer) and MCF‐7 (cancer). Figure 4 shows representative confocal microscopy. B2P and B2PI were studied following 4 h and 17 h incubation in MCF‐7 cells with 10 μM of each BODIPY derivative. A 17 h incubation was selected to allow for adequate cell uptake of the complex, ensuring sufficient brightness for confocal imaging while minimising cell toxicity. MB2P and MB2PI were evaluated under the same conditions but interestingly, despite their greater molecular brightness, the emission intensity from within cells from this complex was too low for imaging at a 10 μM concentration, this may be due to poorer uptake or distribution of the compound into aqueous regions where it does not emit strongly. However, at a higher concentration of 30 μM used across all subsequent studies the compounds showed strong uptake and emission from within cells.

Across all four compounds uptake of the BODIPYs led to extremely bright and highly punctate staining from within the cells. As all of the BODIPY‐perylene probes emit minimally from aqueous media including cell culture media (Figure S20) the bright emission is attributed to localisation of the complex to non‐aqueous domains, i. e. organelle membranes or to lipid droplets (LDs). To identify which, co‐localisation studies with targeted probes were completed. CHO and MCF‐7 cells were treated with B2P, B2PI, MB2P or MB2PI and co‐stained with specific organelle markers including LD probes; PyLa−C17Cer and BODIPY 493/503, an endoplasmic reticulum stain; ER Tracker Blue‐White and a lysosomal stain; LysoTracker Deep Red. Pyla‐C17Cer was previously published and localises in both the ER and LDs.^[41]^ Cellular uptake of B2PI was recently reported in MCF‐7 cells, though localisation was not explored.^[25]^ Here, we found that B2P, B2PI and MB2P localise strongly to the LDs of both test cell lines where the highest Pearson's coefficients were observed for B2P at 0.78±0.09 and 0.69±0.03 with PyLa−C17Cer and BODIPY 493/503 respectively.

MB2PI also accumulates in LDs of MCF‐7 cells (Pearson's coefficient=0.49 PyLa−C17Cer/0.45 BODIPY 493/503), but its accumulation was comparatively reduced in CHO cells where the Pearson's coefficient for LD probe was <0.3, it tended instead, as reflected in Table S2, to show preference for the ER and lysosome in this cell line. Indeed, all the BODIPY‐perylene dyes tested entered the lysosomes of MCF‐7 cells to varying extents, however only B2PI, MB2P and MB2PI localise in the lysosomes of CHO cells. Finally, B2P showed no affinity for the ER in either test cell line which is not surprising based on its highly punctate staining. A Pearson's coefficient of *ca* 0.5 was observed for B2PI, MB2P and MB2PI with ER Tracker in CHO cells and MB2P and MB2PI in MCF‐7 cells. The co‐localisation results are summarised in Table S2.

Lysosomes are a rational targets for PDT agents as they play an essential role in cellular homeostasis and photosensitised disruption of their function has been demonstrated to lead to cell death, for example Hu et al. reported such an outcome with a lysosomal targeted Aza‐BODIPY.^[42]^ LDs have also been reported as cogent targets for PDT, for example Tabero et al. were the first to report targeting LDs for photodynamic theragnosis, a strategy combining bioimaging and PDT.^[43]^ LDs are closely linked with the ER and emerging evidence indicates that damage to the ER and/or LDs can induce cancer cell death through an ER‐stress mediated process. The ER/LD relationship has been exploited by Chauhan et al. to design a novel BODIPY‐Naphtholimine‐BF_2_ dyad for treating pancreatic cancer.^[44]^ Overall, the localisation of the BODIPY‐perylene derivatives across the lysosomes, ER and LDs of the test cells is desirable both from an imaging perspective and for PDT.

### Emission Spectral Response *in cellulo*

Given the different distributions of MB2PI between cell types seen in confocal imaging and the solvent dependence of the probes, we explored the emission spectroscopy of this compound in the live cellular environment and on stimulation of inflammation which is expected to promote lipid droplet formation. Emission spectra were collected from cells incubated for 17 hours with MB2PI (after washing and exchanging fresh cell media) and show interesting contrasts in the spectroscopy of MB2PI between the two cell lines. Intense emission was observed centred at 650 nm from MB2PI in CHO with a much weaker emission from MCF7 with peaks evident at approximately 540 and 730 nm profile. The origin of these marked differences is interesting and suggests the sensitivity of the photophysical properties to the cell environment, most likely reflecting the variation in subcellular localisation in each cell type. In CHO cells, MB2PI was localised in lysosomes and the ER, but not in LDs. Whereas in MCF‐7 cells, it was found in lysosomes, the ER, and LDs. From solvent studies, in CHO, the data are consistent with a lipophilic environment whereas the emission profile of MCF‐7 is similar to that observed in viscous, more protic environments from solvent/viscosity dependent studies.

We applied TNF‐α as a stimulant for cellular viscosity due to its well‐documented role in promoting inflammation and its known effects on cellular processes, including lipid metabolism.[^45^, ^46^] TNF‐α is known to influence lipid droplet (LD) dynamics by increasing the number and size of lipid droplets within cells. TNF‐α incubation times were varied to explore TNF‐α‐induced changes in LD morphology and this is also expected to influence LD viscosity to which MB2PI may provide a response reflecting inflammatory stimuli. Specifically, cells were pre‐treated with 100 ng/mL TNF‐α for up to 24 h before adding MB2PI.

**Figure 5:**
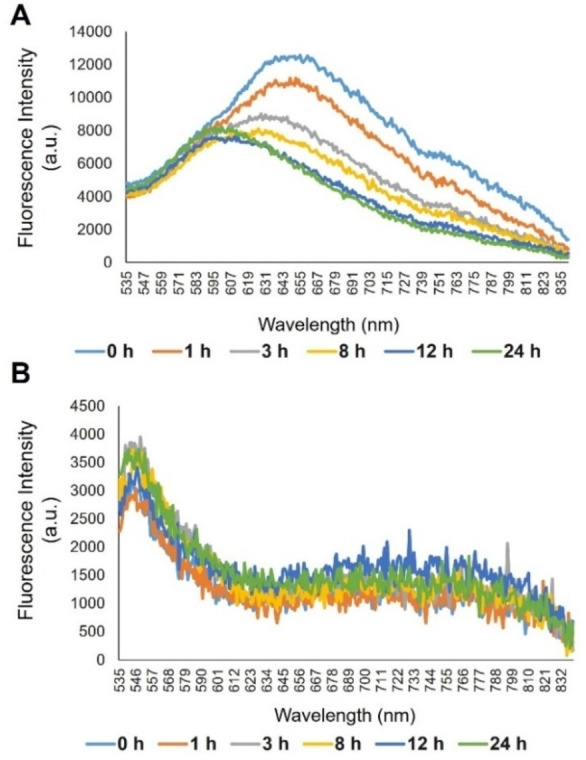
Emission spectral response of MB2PI in (A) CHO and (B) MCF‐7 cells. At 0 h cells were untreated and at subsequent time points cells were pre‐treated with 100 ng/mL TNF‐α for 0–24 h prior to the addition of MB2PI (10 μM, 17 h).

MB2PI spectra in CHO cells (Figure 5A) showed a slight shift and decreased emission after longer incubations with TNF‐α, indicating a potential response to changes in cellular conditions.

BODIPY dyes with meso substituents are known to exhibit viscosity‐sensitive fluorescence.[^39^, ^47^, ^48^, ^49^] We examined the effect of solvent polarity and viscosity on emission spectroscopy of MB2PI in a toluene‐castor oil and glycerol methanol systems as models of highly lipophilic and more protic environments, to see if this might reflect the changes observed on TNF‐α treatment *in cellulo*. The mesityl group blocks rotation at the meso position in this compound but the perylene may act as a rotor. In the toluene‐castor oil system there was an overall blue shift in emission with increasing viscosity though the changes in intensity were not systematic (Figure S21), nonetheless the spectral changes were very similar to TNF‐α treated CHO. In the methanol glycerol system (Figure S22) the emission profile was notably similar to the emission from MCF‐7 cells (Figure 5B). Increasing glycerol concentration led to increased emission intensity but no overall change in emission profile consistent again with the response in MCF7 cells. Therefore, we speculate that the differences in emission profile in each cell type reflect the polarity of the environment the probe is localised to *in cellulo*. As described, MB2PI accumulates in LDs of MCF‐7 cells but primarily to the ER and lysosome in CHO. We would expect the compound to penetrate the membraneless LD in the latter case but from the emission spectroscopy it likely resides in the membranes of the lysosome and ER in CHO. The response to TNF‐α treatment requires further investigation, however the data suggests that simulation of inflammation affects the viscosity membrane. Overall, the data indicates that the environmental sensitivity of the emission spectroscopy of these probes can facilitate interrogation and dynamic tracking of inflammatory response in live cells.

### Toxicity Studies

The family of BODIPY‐perylene probes were found to be minimally toxic in CHO and MCF‐7 cells with an IC_50_ of >50 μM after a 24 h incubation in the dark at 37 °C. Typical of many BODIPY derivatives, the BODIPY's presented in this work are insoluble in aqueous media, including PBS, and thus the main stock solutions (2–3 mM) were prepared in 100 % DMSO and diluted in media immediately before treating the cells. The maximum concentration for dark toxicity was set at 50 μM to keep the concentration of DMSO ≤1 % (v/v) to maintain cell health. As a result, it was not possible to determine an exact dark IC_50_ value as concentrations above 50 μM demanded DMSO concentrations that would affect the viability of the cells. Dietzek‐Ivanšić et al previously reported on the effect of DMSO on MCF‐7 cell viability, where cell viability remained high (95 %) in cells treated with 1 % DMSO in cell culture media for 24 h, whereas only 40 % of cells remained viable after treatment with 3.8 % DMSO. Working stocks in cell culture media remained stable within the incubation times used for cellular studies i. e. up to 24 h.^[25]^

The phototoxicity of the BODIPY's were assessed to compare the light induced toxicity of the iodinated compounds with their non‐iodinated counterparts. CHO and MCF‐7 cells were treated with B2P, B2PI, MB2P, and MB2PI in a concentration range from 0.10–1 μM for 17 h, the wells were decanted and filled with 100 μL phenol red free media prior to irradiation with a 470 nm LED (17 J/cm^2^). Cell viability was determined using the alamar blue cell viability assay and % viability was calculated relative to untreated control wells.

All compounds showed excellent phototoxicity, with PI values in CHO cells ranging of >64 (MB2P), >76 (B2P), >87 (B2PI) and >132 (MB2PI). The BODIPY compounds showed slightly higher phototoxicity in MCF‐7 cells (IC_50_ values in Table 2), however the pattern of least to most phototoxic derivative remained the same where the lowest PI value observed (>109) was in cells treated with MB2P, followed by B2P with a PI value of >182 (B2P). Interestingly, the PI values of B2PI and MB2PI were more similar in MCF‐7 cells than CHO at >213 (B2PI) and >218 (MB2PI). A PI value of between 2 and 5 indicates potential phototoxicity and above 5 indicates phototoxicity, and the PI values reported here surpass this value significantly.^[50]^ These results indicate that the BODIPY‐perylene compounds are potent PSs. Although B2P and MB2P also exhibited high phototoxicity towards the test cells, the substitution of an iodine moiety dramatically enhanced compound phototoxic capacity with B2PI and MB2PI resulting in significantly higher PI values than their non‐iodinated counterparts. This was expected since introduction of the iodine group is expected to promote ISC, leading to greater triplet yields and ^1^O_2_ generation. Investigation into the specific molecular interactions and photophysical properties of the BODIPY compounds B2P and B2PI has been published where the findings conclude that the heavy atom effect and SOCT are additive.^[26]^

**Table 2:** IC_50_ values of CHO and MCF‐7 cells (μM) with and without irradiation.

	Cell Line^[a]^	Dark IC_50_ (μM)	Light IC_50_^[b]^ (μM)	Photo Index^[c]^ (PI)
B2P	CHO MCF‐7 MCF‐7 3D	>50	0.662±80 0.274±60 6.91±0.24	>76 >182 6.6
		46±2.38		
B2PI	CHO MCF‐7 MCF‐7 3D	>50	0.578±50 0.235±21^[d]^ 7.65±0.23	>87 >213 5.7
		44±3.84		
MB2P	CHO MCF‐7 MCF‐7 3D	>50	0.779±50 0.457±13 6.26±2.86	>64 >109 >8
MB2PI	CHO MCF‐7 MCF‐7 3D	>50	0.380±40 0.229±7 5.62±0.21	>132 >218 >8.9

^[a]^ ‘MCF‐7 3D’ indicates MCF‐7 spheroids, ^[b]^ Cells were irradiated at 17±1.64 J/cm^2^ (2.5 mW/cm^2^ for 2 h) and spheroids at 5±0.54 J/cm^2^ (2.2 mW/cm^2^ for 0.5 h), ^[c]^ Phototoxic index (PI) is the ratio of dark IC_50_ (without irradiation) to light IC_50_ (irradiated cells), ^[d]^ B2PI toxicity in MCF‐7 data published previously.^[25]^

Surprisingly, the addition of a mesityl group to MB2P did not result in enhanced phototoxicity, with higher PI values observed for B2P.

Nonetheless, the BODIPY‐perylene derivatives reported here, particularly MB2PI, hold promise for potential applications in targeted photodynamic therapy for diseases such as cancer, as exemplified by their effectiveness in MCF‐7 and CHO cells.

### Mechanism of Phototoxicity

**Figure 6:**
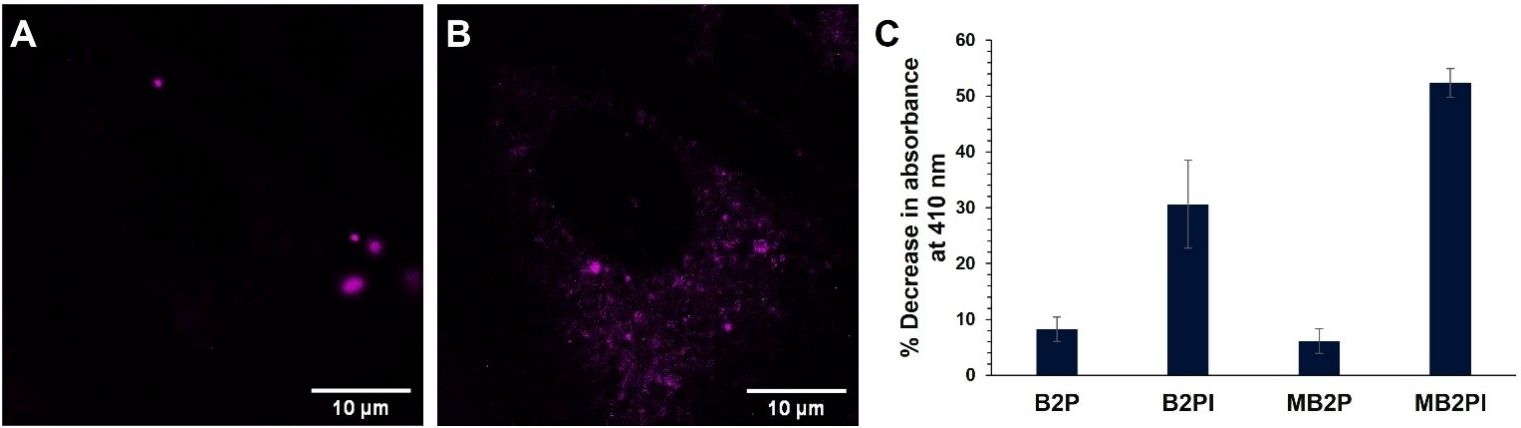
ROS dye H_2_DCFDA (A) prior to irradiation and (B) after 5 minutes of continuous irradiation confirming ROS generation. Cells were treated with MB2PI prior to irradiation (30 μM, 17 h). H_2_DCFDA was excited at 494 nm and emission collected between 517–527 nm. A graph of ROS scavenger DPBF response to irradiation of the BODIPY‐perylene compounds is shown in (C) where a decrease in absorbance at 410 nm confirms ^1^O_2_ generation upon photoactivation. Studies were performed with the BODIPY‐perylene compounds under absorbance matched conditions. Values shown indicate the % decrease in absorbance values recorded at t=0 and t=210 seconds and is reported as mean±standard deviation. Individual results were recorded every 30 seconds from t=0–210 seconds and are displayed in Figure S32.

Given the impact of the heavy iodine atom on phototoxicity, to confirm if the origin of toxicity is triplet sensitised formation of ^1^O_2_ we carried out ROS scavenger studies with 1,3‐diphenylisobenzofuran (DPBF, Figure 6C). Stock solutions of BODIPY‐perylene were prepared under absorbance matched conditions and a BODIPY‐DPBF solution prepared at concentration of 10 mM DPBF in methanol. The BODIPY‐DPBF solution was irradiated (470 nm) from 0–210 seconds and the absorption spectra recorded every 30 seconds. In the presence of ^1^O_2_, DPBF forms an unstable peroxide, endoperoxide 1, that readily decomposes to colourless 1,2‐dibenzoylbenzene (DBB).^[51]^ This change is detected spectrophotometrically with depletion of DPBF absorbance at 410 nm. The DPBF assay results are represented in Figure 6C as % decrease in absorbance at 410 nm. The values are determined at t=210 seconds relative to the absorbance values recorded prior to irradiation at t=0. These results correlate well with the phototoxicity data *in cellulo*. As expected, the most phototoxic compound, MB2PI, generates the most ^1^O_2_ upon irradiation with a 470 nm LED with a 52 % decrease in DPBF absorbance at 410 nm. This is closely followed by a 31 % decrease of DPBF absorbance when irradiated in the presence of B2PI, which also displayed high phototoxicity in the test cells. DPBF is known to be sensitive to light, therefore, to mitigate photodegradation of affecting the assay results, exposure was limited to 3.5 minutes where DPBF absorbance decrease at 410 nm is <10 % at this time point. The decrease in absorbance of DPBF alone upon irradiation by the 470 nm LED suggests that B2P and MB2P are not generating ROS after 3.5 minutes of irradiation, rather the DPBF itself is degrading as a decrease in absorbance of only 8 % (B2P) and 6 % (MB2P) were observed for these derivatives. Due to the photo‐vulnerability of DPBF under visible irradiation, this study is limited by a short experimental window and ignores any ROS generation over longer irradiation periods. It is important to note that although DPBF is highly sensitive to ^1^O_2_, it can also react with other ROS, for example, superoxide anions or hydroxyl radicals.^[51]^

To study the production of ROS in a live cell environment a commercial ROS dye, H_2_DCFDA was used. CHO and MCF‐7 cells were incubated with MB2PI (30 μM, 17 h), washed with PBS and co‐stained with H_2_DCFDA (5 μM, 30 min). To ensure no photoactivation of MB2PI, emission from H_2_DCFDA was collected alone prior to the excitation of the BODIPY. The complex was then irradiated continuously at 488 nm (0.38 mW/cm^2^) for 5 minutes. As shown in Figure 6, irradiating MB2PI results in increased production of ROS in CHO cells with extensive cytoplasmic staining, though no ROS production was observed in the nucleus. Weak emission of H_2_DCFDA was observed prior to irradiation, consistent with the low cytotoxicity of the complex. However, after irradiation, the fluorescence intensity of the ROS dye increases dramatically indicating ROS production, consistent with the high phototoxicity of MB2PI in both test cell lines. The higher phototoxicity of MB2PI in MCF‐7 cells restricted the ROS imaging study to CHO cells as the MCF‐7 cells began to die rapidly upon irradiation. Controls completed by adding H_2_DCFDA in test cells without MB2PI confirmed no ROS production with or without irradiation at 488 nm.

As MB2PI was found to be the most phototoxic BODIPY under normoxic conditions, it was selected for further analysis under chemically induced hypoxia to ascertain the role of oxygen in the phototoxicity of the BODIPYs. Cobalt chloride (CoCl_2_), a popular chemical hypoxia inducer, was added to the cells (after incubation with B2PI or MB2PI) at a total concentration of 300 μM CoCl_2_ in media and incubated for 4 h to mimic hypoxic conditions. In comparison to low‐oxygen induced hypoxia, this method stabilises hypoxia inducible factor 1 alpha subunit (HIF‐1α) for some hours after treatment, allowing a greater window for the phototoxicity studies.^[52]^ We have previously confirmed the chemical induction of hypoxia with CoCl_2_ using an enzyme linked immunosorbent assay to detect HIF‐1α in cell lysates.^[53]^ The cells were irradiated immediately upon removal of the media/CoCl_2_ solution as per the normoxic phototoxicity protocol. As expected, phototoxicity was significantly reduced under hypoxic conditions, with good cell viability maintained up to our maximum condition of 1 μM i. e. hypoxic IC_50_ is >1 μM, in contrast to the light IC_50_ values of <0.6 μM and *ca*. 0.2 μM for the BODIPYs tested under normoxic conditions in CHO and MCF‐7 cells respectively, confirming the pathway is likely Type II.

### 3D Cell Studies

**Figure 7:**
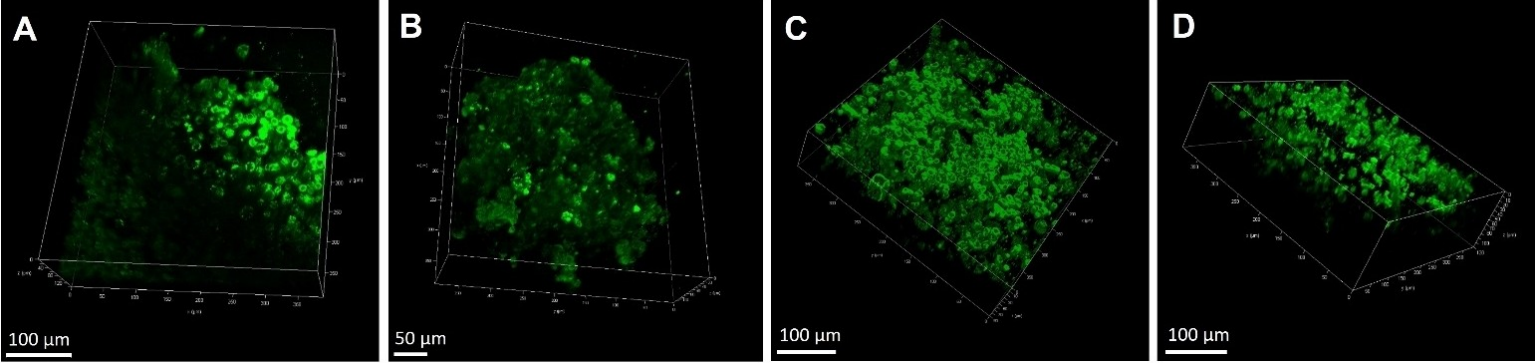
3D reconstruction of live MCF‐7 spheroids treated with (A) B2P, (B) B2PI, (C) MB2P or (D) MB2PI (50 μM/17 h). Z‐stack images were captured from below to above the spheroids and combined to produce the 3D reconstructions. Scale bars read 100 μm (A, C, D) and 50 μM (B).

To investigate if the uptake of the BODIPY probes carried from cell monolayers to 3D cell models, the distribution of B2P and MB2PI was assessed in a tumour‐like environment. Multicellular tumour spheroids are a popular choice of 3D cell model and provide a more physiologically relevant model to study potential PSs. Spheroids are a superior model of *in vivo* tumour compared to cell monolayers. They comprise a layered organisation with an outer proliferative zone, a senescent or quiescent zone and often a hypoxic or necrotic core which is analogous particularly to metastatic tumours.^[54]^ Additionally, there are increased cell‐to‐cell and cell‐to‐extracellular matrix interactions that produce physical barriers, better reflecting potential drug uptake in a real tumour.^[55]^ To assess uptake of the BODIPYs in MCF‐7 spheroids, the BODIPY‐perylene compound were incubated with the spheroids at 50 μM for 17 h and imaged using a confocal microscope. All the BODIPY derivatives were found to deeply penetrate MCF‐7 spheroids as shown in representative imaging in Figure 7, where there is widespread distribution of B2P and B2PI throughout the depth of the spheroid. Interestingly, consistent with 2D cell culture, MB2P and MB2PI were not taken up throughout the spheroid as effectively as B2P and B2PI. Consistent also with 2D studies all compounds were nuclear excluded and evident from punctate features widespread in the cytoplasm. Notably, the dyes exhibited strong fluorescence in the spheroids, requiring a lower laser intensity for imaging than cell monolayers. In spite of the evidently lower uptake, phototoxicity studies confirmed MB2PI is the most phototoxic BODIPY‐perylene derivative in MCF‐7 spheroids, with a PI of >8.9. It is important to note that studies in spheroids were performed at a lower irradiation dose of 5 J/cm^2^, as spheroid viability was affected at 17 J/cm^2^. Generally, PI values are significantly lower in spheroids in comparison to studies in cell monolayers.[^53^, ^56^] Nonetheless, the PI values reported are notable and indicate effective phototoxicity in a tumour‐like environment by these compounds. The data also underscore the need for 3D models as an intermediate between studies in cell monolayers and *in vivo* studies. Given that spheroids are an underexplored model for BODIPY applications, the difference in phototoxicity of the BODIPY‐perylene derivatives observed in cell monolayers compared to spheroids highlights the importance of further investigation into their use for more accurate and clinically relevant assessments of novel BODIPY‐based PSs for PDT.

## Experimental Section

### Synthesis of Mesityl‐BODIPY (1)

Mesitaldehyde (0.5 mL, 3.39 mmol) was dissolved in 2 mL of dichloromethane and 2,4‐dimethylpyrrole (0.74 mL, 7.12 mmol, 2.1 eq.) and 5 drops of TFA were added. The reaction was stirred at RT for 6 hours and *p*‐chloranil (917 mg, 3.73 mmol, 1.1 eq.) was added dissolved in 10 mL of DCM. After one 1 hour, trimethylamine (2.84 mL, 20.34 mmol, 6 eq.) and BF_3_ etherate (3.35 mL, 27.12 mmol, 8 eq.) were added and the reaction stirred at room temperature overnight. Solvents were evaporated under vacuum and the product was purified by column chromatography using cyclohexane ethyl acetate (9 : 1) to yield a red solid in a 32 % yield. ^1^H‐NMR (CDCl_3_, 600 MHz): *δ* (ppm) 6.94 (s, 2H), 5.95 (s, 2H), 2.55 (s, 6H), 2.33 (s, 3H), 2.09 (s, 6H), 1.38 (s, 6H).

### Synthesis of Monobrominated Mesityl‐BODIPY (2)

Mesityl‐BODIPY (908 mg, 2.48 mmol) was dissolved in 20 mL of THF; NBS (441 mg, 2.48 mmol, 1 eq.) was added in small fractions over a period of 20 minutes. The reaction was stirred at room temperature for 1 hours. Solvents were evaporated under vacuum and the product recrystallised from methanol. Addition of ice facilitated the crystallisation. The crystals were filtered under vacuum to afford monobrominated mesityl‐BODIPY in 84 % yield and with good purity. ^1^H‐NMR (CDCl_3_, 600 MHz): *δ* (ppm) 6.95 (s, 2H), 6.01(s, 1H), 2.58 (s, 3H), 2.56 (s, 3H), 2.33 (s, 3H), 2.07 (s, 6H), 1.39–1.36 (m, 2H).

### Synthesis of MB2P

Monobrominated mesityl‐BODIPY **2** (140 mg, 0.314 mmol), 3‐perylene boronic acid pinacol ester (143 mg, 0.377 mmol, 1.2 eq.), K_3_PO_4_ (134 mg, 0.629 mmol, 2 eq.) and XPhos Pd G4 (13.5 mg, 0.015 mmol, 0.05 eq.), were dissolved in 3 mL of THF/water (9 : 1) and the reaction was stirred at 40 °C for 48 hours. Solvents were evaporated under vacuum and the product was purified by column chromatography using silica gel and cyclohexane/ethyl acetate (9 : 1) to afford MB2P in a 54 % yield. ^1^H‐NMR (CDCl_3_, 600 MHz): *δ* (ppm) 8.24–8.15 (m, 4H), 7.67 (d, ^3^ *J*=8.58), 7.50–7.46 (m, 2H), 7.46–7.39 (m, 2H), 7.29 (d, ^3^ *J*=8.58), 6.95 (s, 2H), 6.03 (s, 1H), 2.62 (s, 3H), 2.43 (s, 3H), 2.31 (s, 3H), 2.21 (s, 3H), 2.19 (s, 3H), 1.43 (s, 3H), 1.22 (s, 3H). ^13^C‐NMR (CDCl_3_, 150 MHz): *δ* (ppm) 155.7, 154.5, 142.8, 142.0, 140.0, 138.8, 135.04, 134.98, 134.8, 134.1, 131.7, 131.43, 131.39, 131.24, 131.15, 130.5, 129.4, 129.22, 129.18, 129.1, 128.7, 128.04, 128.0, 126.9, 126.74, 126.73, 126.0, 121.2, 120.51, 120.48, 120.3, 119.9, 21.3, 19.81, 19.77, 14.8, 13.6, 13.5, 11.9. HR‐MS (ESI‐TOF) *m*/*z*: calculated for C_42_H_35_BF_2_N_2_ 616.2863 found 616.2872.

### Synthesis of MB2PI

MB2P (85 mg, 0.137 mmol) was dissolved in 5 mL of dichloromethane and recrystallised NIS (37.22 mg, 0.165 mmol, 1.2 eq.) the reaction was stirred at room temperature for 16 hours. The solvent was evaporated under vacuum and the product purified by column chromatography to afford MB2PI as a dark red solid in 73 % yield. ^1^H‐NMR (CDCl_3_, 600 MHz): *δ* (ppm) 8.24–8.17 (m, 4H), 7.69 (d, 2H), 7.71–7–66 (m, 2H), 7.51–7.46 (m, 2H), 7.46–7.42 (m, 1H), 7.39–7.35 (m, 1H), 7.28 (d, ^3^ *J*=7.64), 6.97 (s, 2H), 2.69 (s, 3H), 2.43 (s, 3H), 2.32 (s, 3H), 2.19 (s, 3H), 2.17 (s, 3H), 1.46 (s, 3H), 1.21 (s, 3H). ^13^C‐NMR (CDCl_3_, 150 MHz): *δ* (ppm) 157.0,154.8, 142.9, 142.0, 141.9, 139.1, 135.0, 134.9, 134.8, 134.0, 132.6, 131.8, 131.4, 131.3, 131.2, 131.1, 131.0, 130.8, 130.6, 129.4, 129.35, 129.31, 129.1, 128.7, 128.2, 128.1, 127.0, 126.79, 126.75, 125.7, 120.6, 120.55, 120.47, 119.9, 84.3, 21.3, 19.85, 19.80, 16.0, 15.7, 13.7,12.2. HR‐MS (ESI‐TOF) *m*/*z*: calculated for C_42_H_33_BF_2_IN_2_ 741.1749 found 741.1762.

### Crystallography of MB2P

The data were collected at 100(1) K on a Synergy, Dualflex, AtlasS2 diffractometer using Cu*K*α radiation (λ=1.54184 Å) and the *CrysAlis PRO* 1.171.40.67a suite.^[57]^ Using SHELXLE^[58]^ and Olex2^[59]^ the structure was solved by dual space methods (SHELXT^[60]^) and refined on *F* ^2^ using all the reflections (SHELXL‐2018/3^[61]^). All the non‐hydrogen atoms were refined using anisotropic atomic displacement parameters, except for the carbon atoms of the overlapping, partial‐occupancy chloroform solvate molecules and the 5 % occupancy chlorine atoms. SAME restraints were applied to all the perylene groups, to the disordered section of the BODIPY in mol 1, and to the chloroform molecules. Hydrogen atoms were inserted at calculated positions using a riding model. The crystals diffracted poorly and, despite a long collection time (*ca*. 80 hr), the data set is very weak and the structure shows significant disorder. Consequently, the precision of the structure determination is reduced but the main features are clear.

**Crystal Data** for C_42.475_H_35.475_BCl_1.425_F_2_N_2_ (*M*=673.23 g/mol): monoclinic, space group P2_1_/c (no. 14), *a*=22.5919(8) Å, *b*=12.5812(5) Å, *c*=25.2321(9) Å, *β*=96.352(3)°, *V*=7127.8(5) Å^3^, *Z*=8, *T*=100.01(11) K, μ(Cu Kα)=1.584 mm^−1^, *D_calc_* =1.255 g/cm^3^, 52467 reflections measured (7.05°≤2θ≤133.202°), 12557 unique (*R*_int_=0.0886, R_sigma_=0.0612) which were used in all calculations. The final *R*_1_ was 0.1039 (I>2σ(I)) and *wR*_2_ was 0.3148 (all data). CCDC 2157191 contains the supplementary crystallographic data for this paper. These data can be obtained free of charge from The Cambridge Crystallographic Data Centre via www.ccdc.cam.ac.uk/data_request/cif.

### Live Cell Imaging

MCF‐7 and CHO cells were treated with B2P, MB2P, B2PI or MB2PI for 17 h and co‐stained with a number of dyes for co‐staining including LysoTracker Deep Red, ER Tracker Blue/White, PyLa−C17Cer, BODIPY 493/503 and H_2_DCFDA. Cells were imaged in phenol red free media using a Leica DMi8 confocal microscope with a 63x or 100x oil immersion lens and a heated state at 37 °C.

### Toxicity Studies

Dark‐ and photo‐toxicity studies were performed by seeding CHO and MCF‐7 cells in clear 96‐well plates (Nunc) and treating with varying concentrations of B2P, MB2P, B2PI and MB2PI. Phototoxicity experiments were carried out using a 470 nm LED. For hypoxic phototoxicity studies, hypoxia was mimicked by adding CoCl_2_ to the cells prior to irradiation. Cell viability was determined using the alamar blue assay and absorbance measured with a BMG LABTECH CLARIOstar plate reader.

## Conclusions

The uptake and phototoxicity of a family of BODIPY‐perylene charge transfer compounds was studied. The de‐novo synthesis of BODIPY‐perylene containing mesitylated units; MB2P and MB2PI is reported, and the crystal structure of MB2P is defined. The photophysics of these compounds, their cellular uptake and phototoxicity in 2D and 3D *in vitro* cell models were studied. All compounds are readily permeable to CHO and MCF‐7 cells and localise mainly in the LDs and lysosomes, common localisation for BODIPY derivatives, that is associated with their lipophilicity. All compounds showed excellent phototoxicity. Under 17 J/cm^2^ irradiation (470 nm LED) B2P, B2PI, MB2P and MB2PI provide impressive PI values of >109 (MB2P), >182 (B2P), >213 (B2PI) and >218 (MB2PI) in MCF‐7 cells and >64 (MB2P), >76 (B2P), >87 (B2PI) and >132 (MB2PI) in CHO cells. Correlations between the DPBF assays and phototoxicity indices indicate that ROS, most likely ^1^O_2_ formation, is responsible for this effect in B2PI and MB2PI since functionalisation with iodine increased phototoxicity in both test cell lines. The most toxic complex, MB2PI, with a PI value of >132 and >218 in CHO and MCF‐7 cells respectively was almost twice that of its non‐iodinated counterpart. These results confirm the combination of charge transfer and iodination can be employed to promote triplet state formation effectively enhancing phototoxic capacity. Furthermore, phototoxicity and ROS generation by the MB2PI is superior to that of B2PI, likely attributed to the greater fluorescent yield and lifetime of the mesitylated compound. Notably, increased phototoxicity was not associated with increased dark toxicity up to 50 μM, with the IC_50_ of our iodinated BODIPY's remaining >50 μM.

The ROS generating capability of MB2PI was confirmed *in cellulo* by imaging the cells with ROS dye H_2_DCFDA prior and post irradiation. Photoirradiation of MB2PI in cells under hypoxic conditions further confirmed this with a marked decrease in phototoxicity, confirming phototoxicity is oxygen dependent. It is important to note that the reported PI values are likely much higher than calculated as the exact dark IC_50_ could not be established due to solubility limitations of the dyes in water. Nonetheless, these results highlight the promise of the BODIPY‐perylene derivatives presented here as powerful tools for PDT. Confocal imaging of MCF‐7 spheroids confirmed the BODIPY probes can penetrate a 3D tumour model, though MB2P and MB2P fail to penetrate throughout the entire spheroid. Despite this, nonetheless impressive PI values >8.9 were observed in MCF‐7 spheroids, where MB2PI was again found to be the most phototoxic with a PI value of >8.9.

## Supporting Information Summary

The authors have cited additional references within the Supporting Information.[^57^, ^58^, ^59^, ^60^, ^61^]

CCDC 2157191 contains the supplementary crystallographic data for this paper. These data can be obtained free of charge from The Cambridge Crystallographic Data Centre via www.ccdc.cam.ac.uk/data_request/cif.

## Conflict of Interests

The authors declare no conflict of interest.

1

## Supporting information

As a service to our authors and readers, this journal provides supporting information supplied by the authors. Such materials are peer reviewed and may be re‐organized for online delivery, but are not copy‐edited or typeset. Technical support issues arising from supporting information (other than missing files) should be addressed to the authors.

Supporting Information

## Data Availability

The data that support the findings of this study are available from the corresponding author upon reasonable request.
