# Supplementary material for: Enhancing Phototoxicity in BODIPY‐Perylene Charge Transfer Dyads by Combined Iodination and Mesylation
Source: Chemistry. 2024 Nov 9;30(71):e202403149. doi: 10.1002/chem.202403149 (PMC11653243; doi:10.1002/chem.202403149)
Supplement: Supplementary file 1 — Supporting Information [file CHEM-30-e202403149-s001.pdf]

# Chemistry–A European Journal

Supporting Information


## Enhancing Phototoxicity in BODIPY-Perylene Charge Transfer Dyads by Combined Iodination and Mesylation


Rhianne C. Curley, Ruben Arturo Arellano-Reyes, James N. McPherson, Vickie McKee, and Tia E. Keyes*

# Electronic Supporting Information

# Enhancing Phototoxicity in BODIPY-perylene Charge Transfer Dyads by Combined Iodination and Mesylation.

Rhianne C. Curley,[a] Ruben Arturo Arellano-Reyes,[a] James N. McPherson,[b] Vickie McKee,[b] and Tia E. Keyes* [a]

a. School of Chemical Sciences, National Centre for Sensor Research, Dublin City University, Glasnevin, Dublin 9, Ireland
b. Department of Physics, Chemistry and Pharmacy, University of Southern Denmark, Campusvej 55, 5230 Odense M, Denmark

* *Corresponding author*: *tia.keyes@dcu.ie*

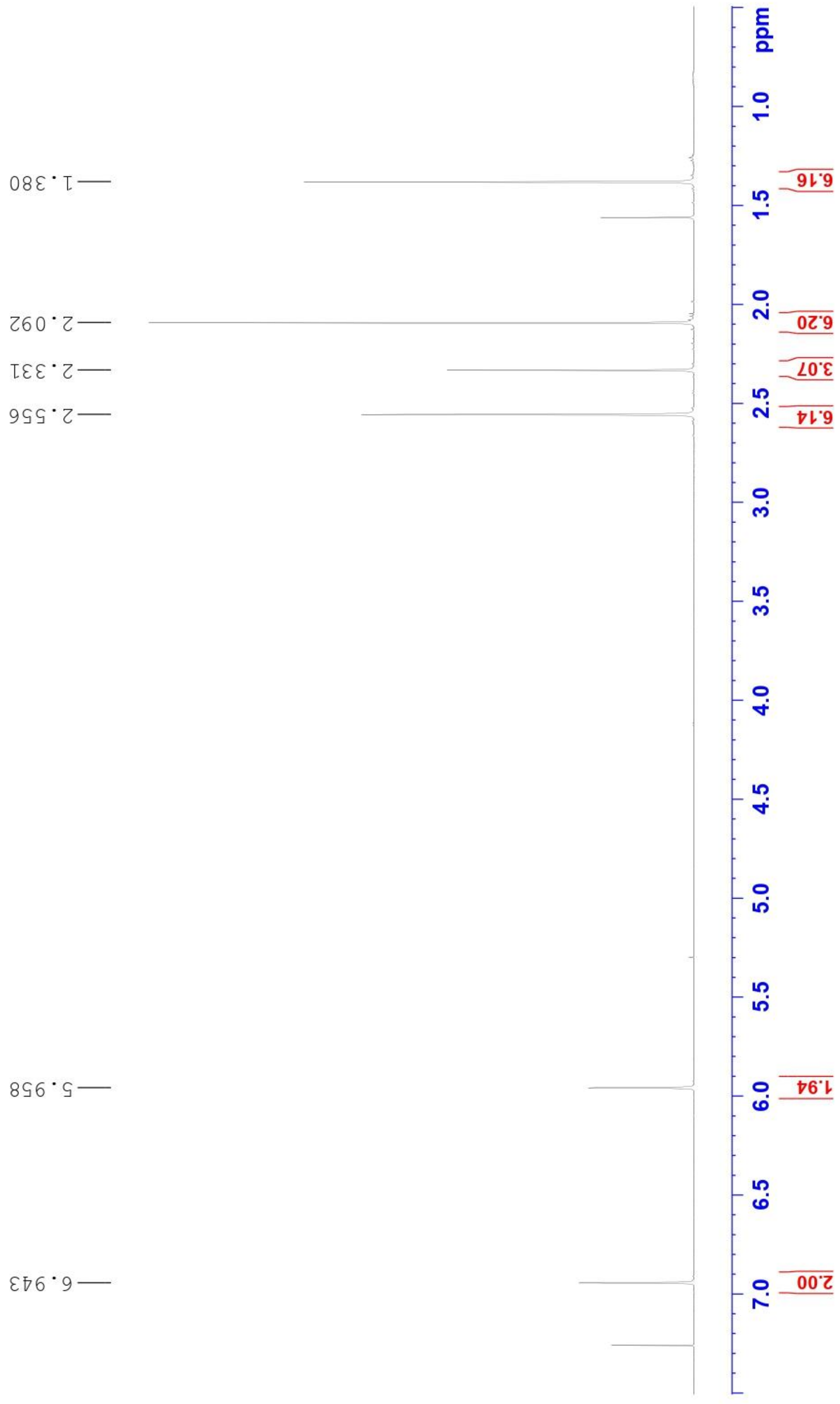


Figure S1. $^{1}$H-NMR spectra of Mesityl BODIPY in $CDCl_3$ at 600 MHz.

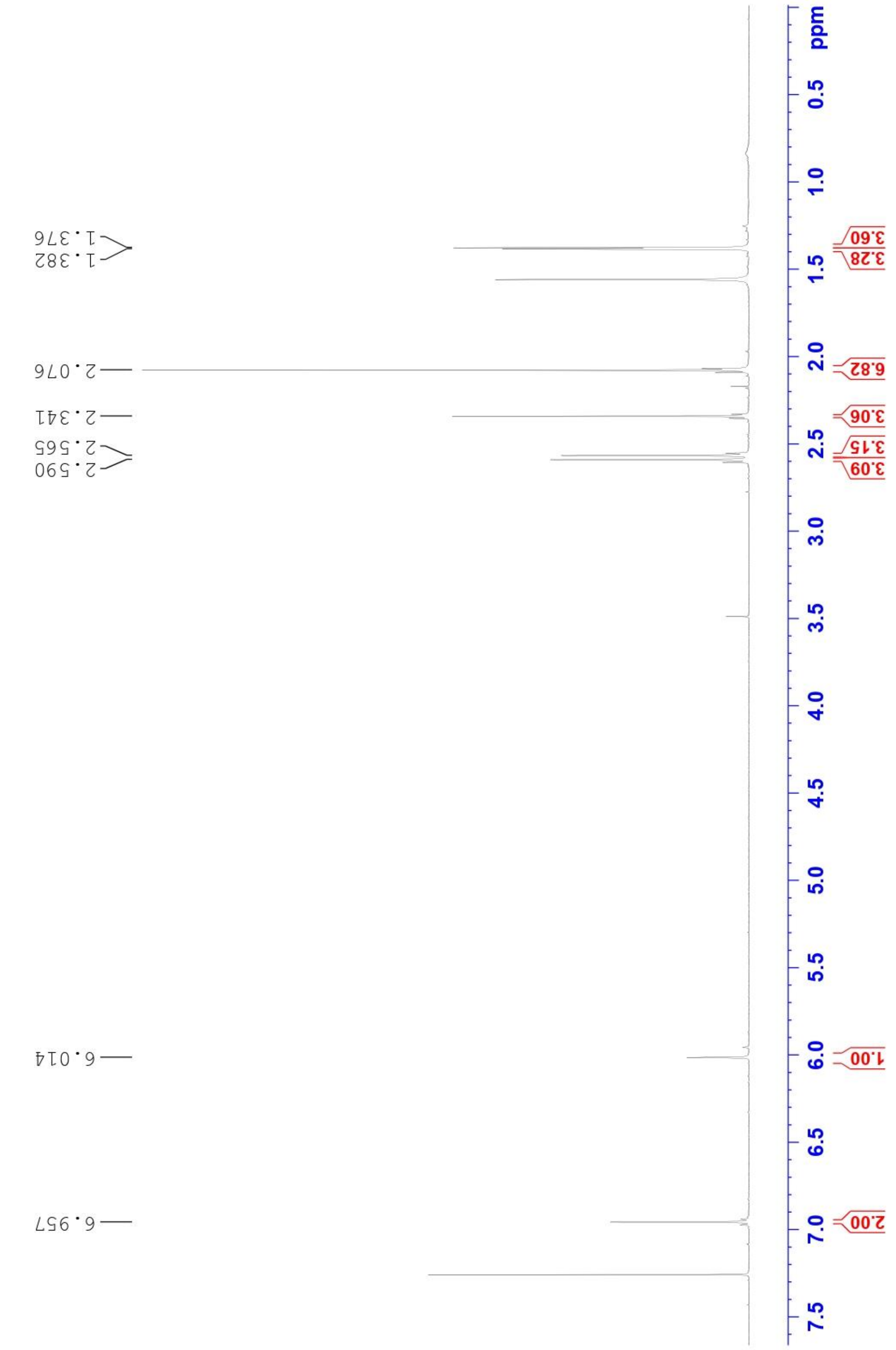


Figure S2. $^{1}H$-NMR spectra of 2-BrMesityl BODIPY in $CDCl_3$ at 600 MHz.

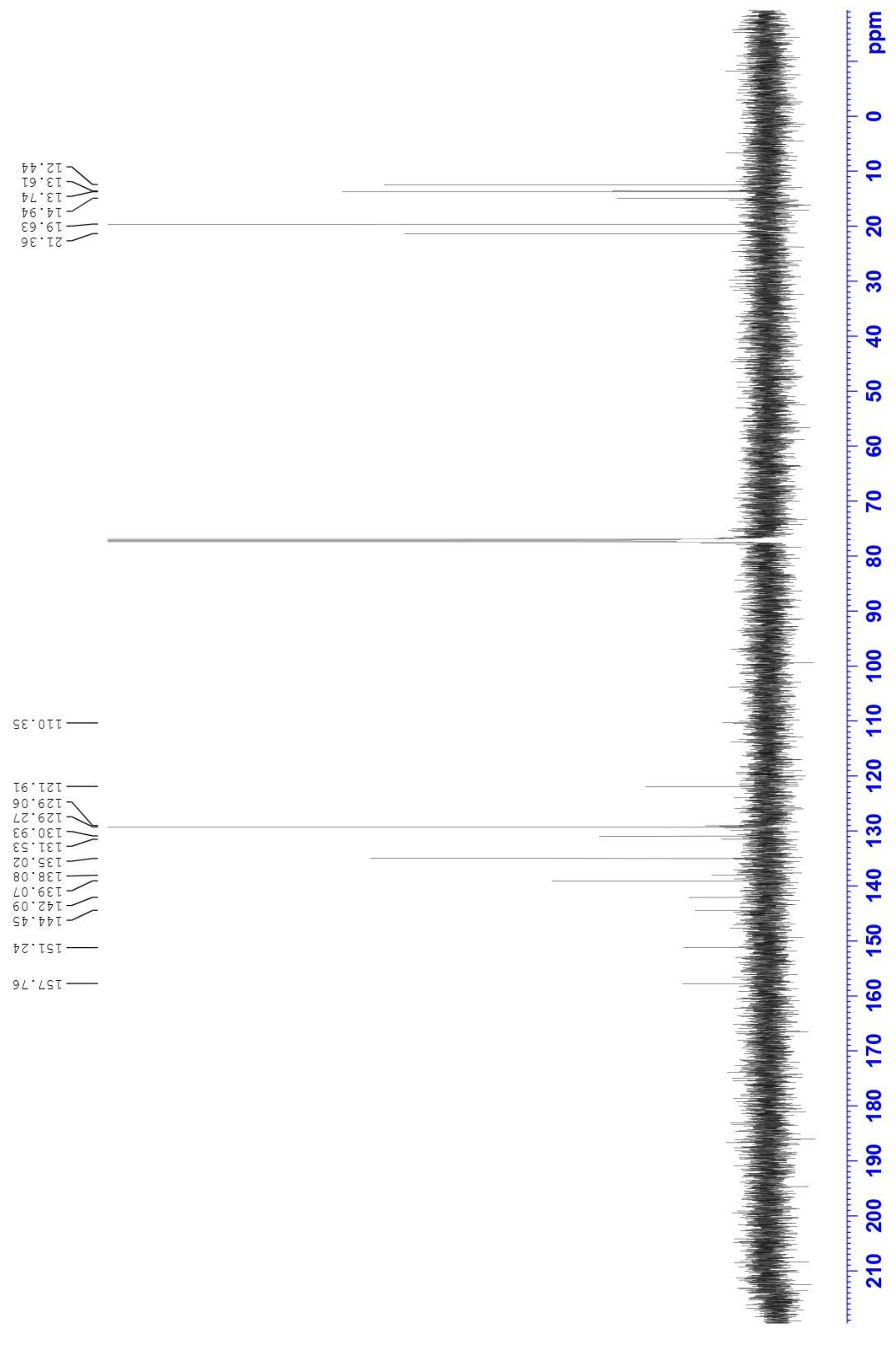


Figure S3. $^{13}$C-NMR spectra of 2-BrMesityl BODIPY in $CDCl_3$ at 150 MHz.

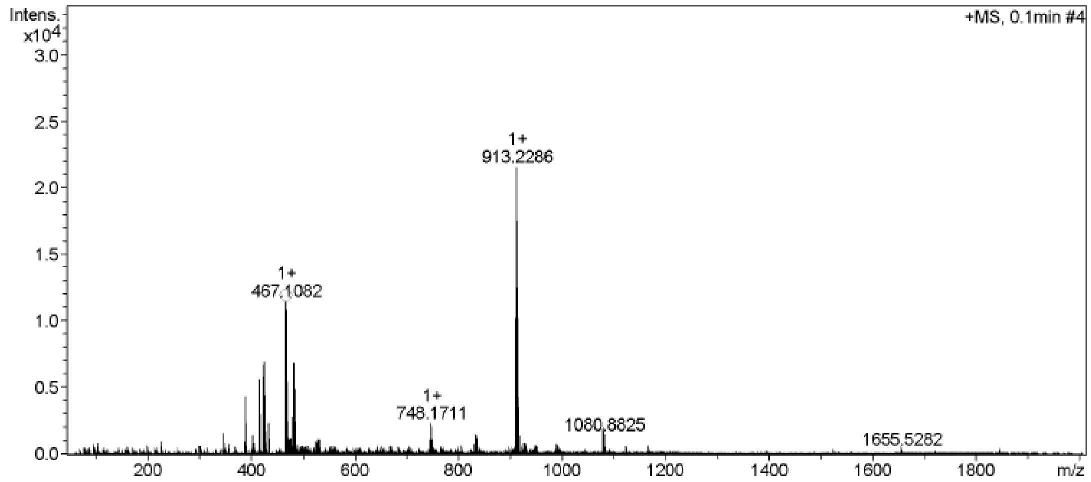


| Meas. m/z | # | Ion Formula | m/z | err [mDa] | err [ppm] | rdb | N-Rule | e⁻ Conf | mSigma |
|---|---|---|---|---|---|---|---|---|---|
| 467.108163 | 1 | C22H24BBrF2N2Na | 467.108015 | -0.1 | -0.3 | 10.5 | ok | even | 42.6 |

Figure S4. HR-MS (ESI-TOF) positive scan of 2-BrMesityl BODIPY.

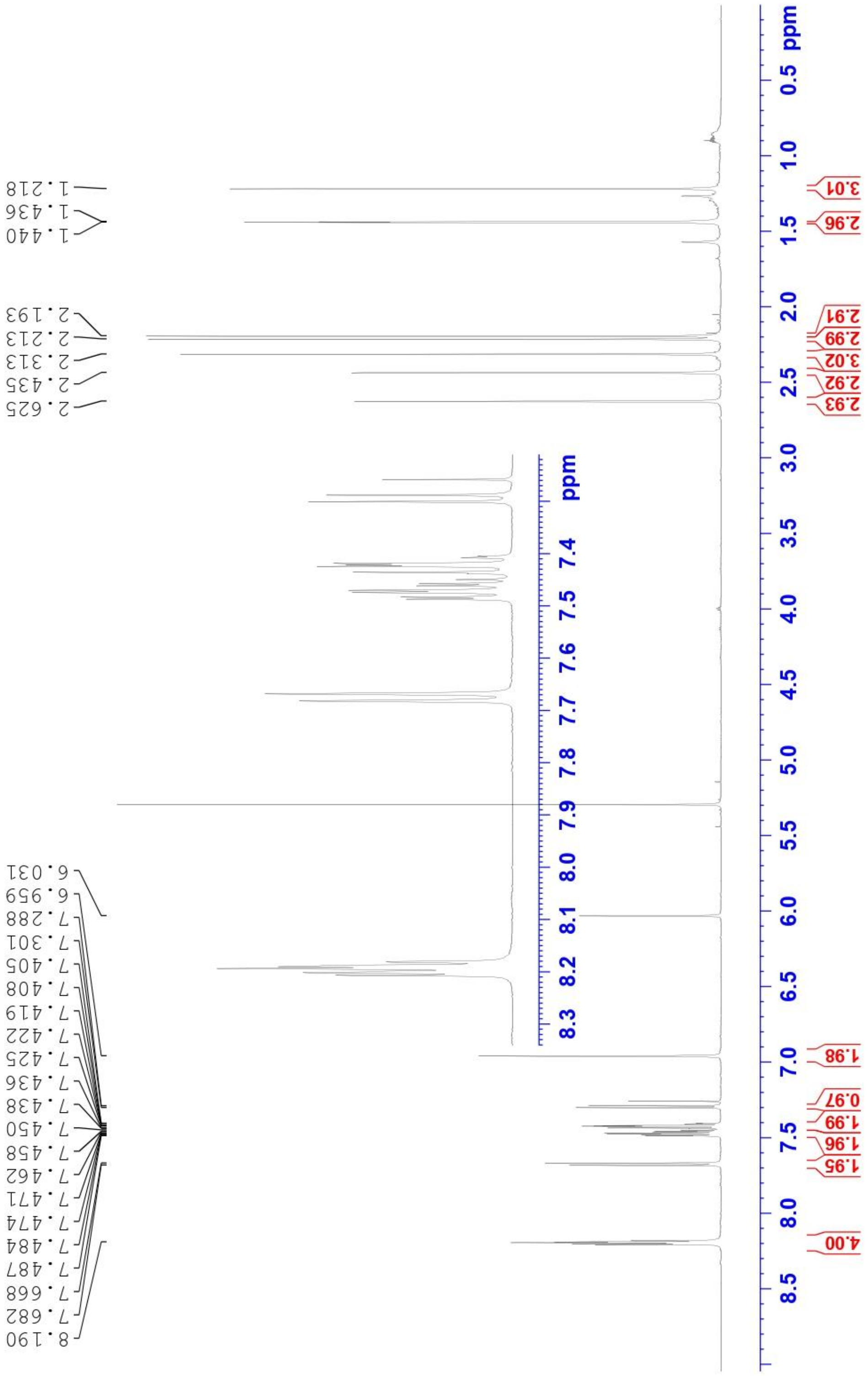

Figure S5. $^1$H-NMR spectra of MB2P in $CDCl_3$ at 600 MHz

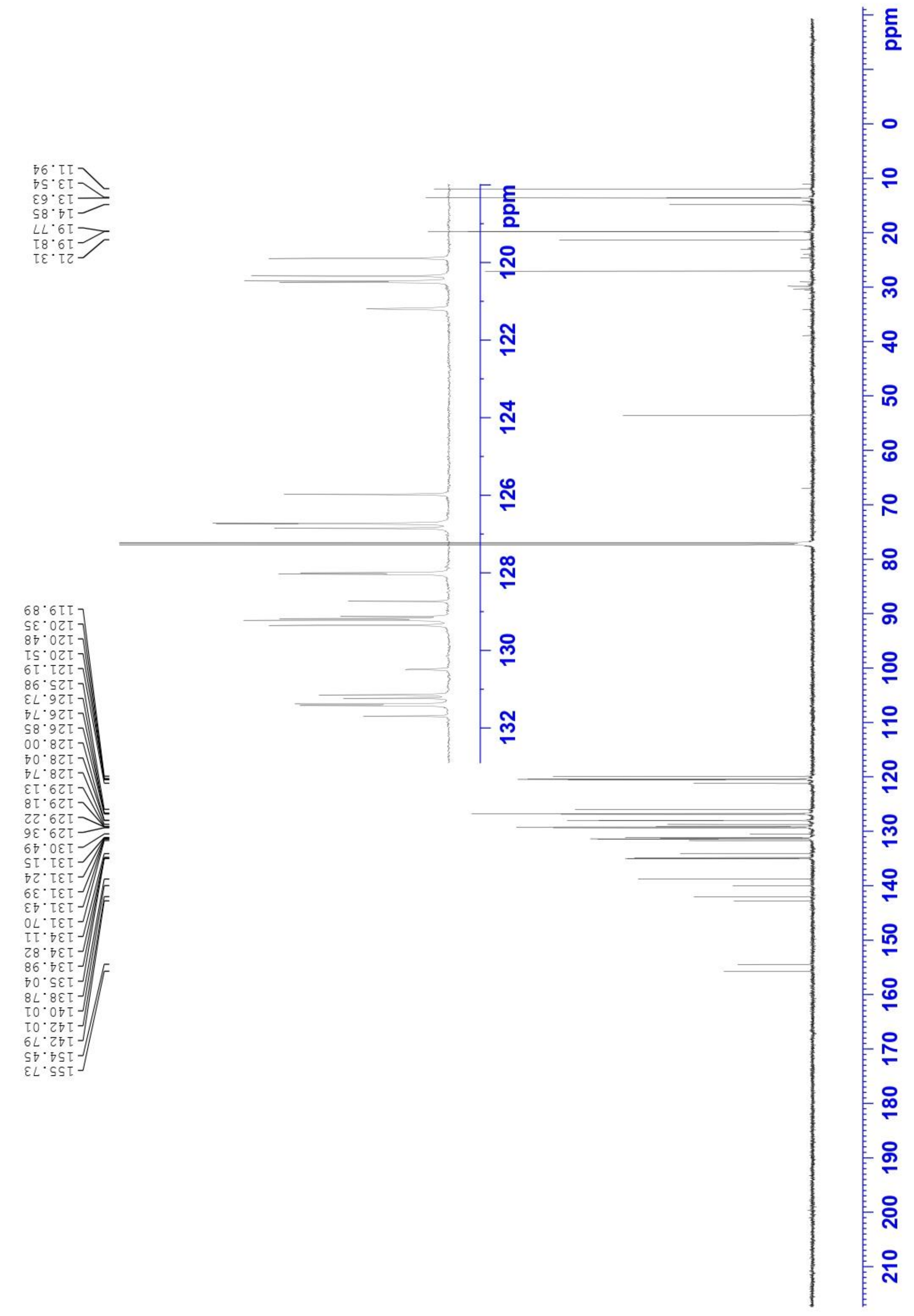


Figure S6. $^{13}$C-NMR spectra of MB2P in $CDCl_3$ at 150 MHz

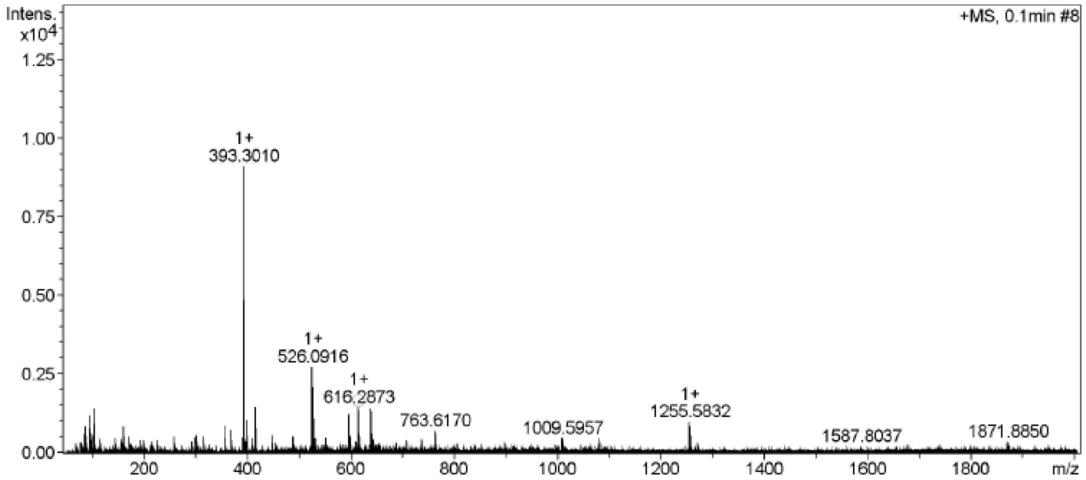


| Meas. m/z | # | Ion Formula | m/z | err [mDa] | err [ppm] | rdb | N-Rule | e⁻ Conf | mSigma |
|---|---|---|---|---|---|---|---|---|---|
| 616.287277 | 1 | C42H35BF2N2 | 616.286304 | -1.0 | -1.6 | 26.0 | ok | odd | 25.9 |

Figure S7. HR-MS (ESI-TOF) positive scan of MB2P.

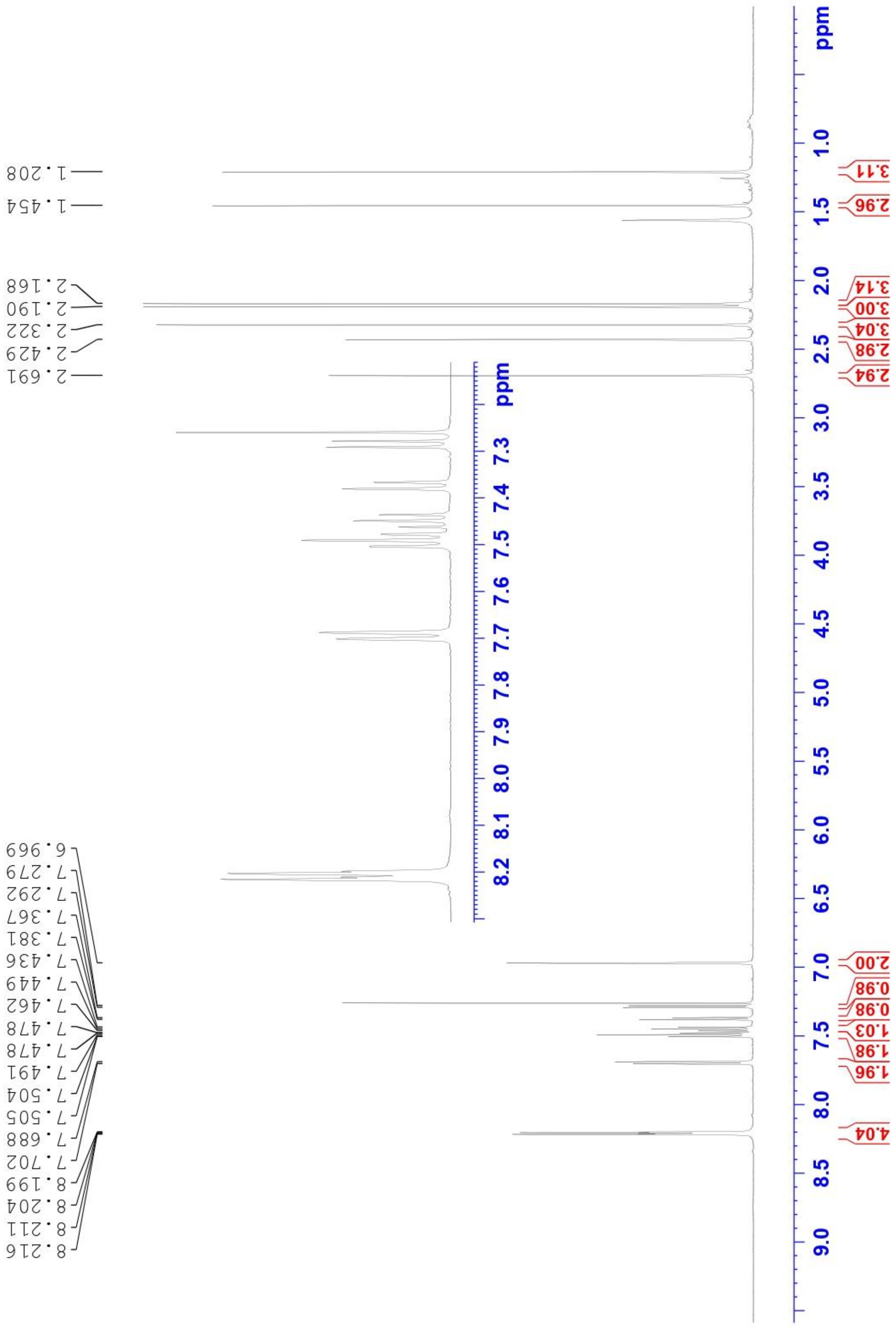


Figure S8. $^{1}H$-NMR spectra of MB2PI in $CDCl_3$ at 600 MHz

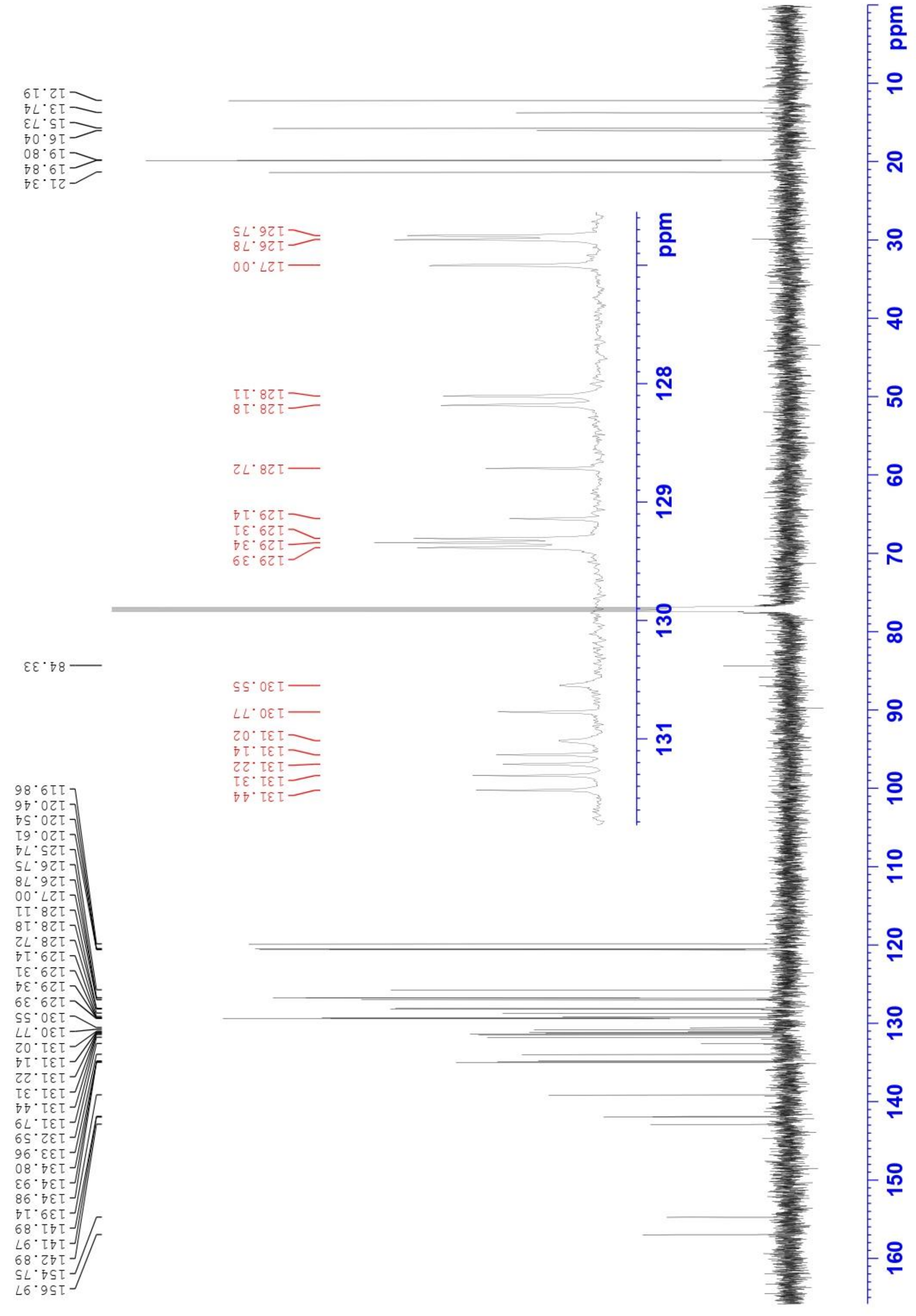


Figure S9. $^{13}$C-NMR spectra of MB2PI in $CDCl_3$ at 150 MHz.

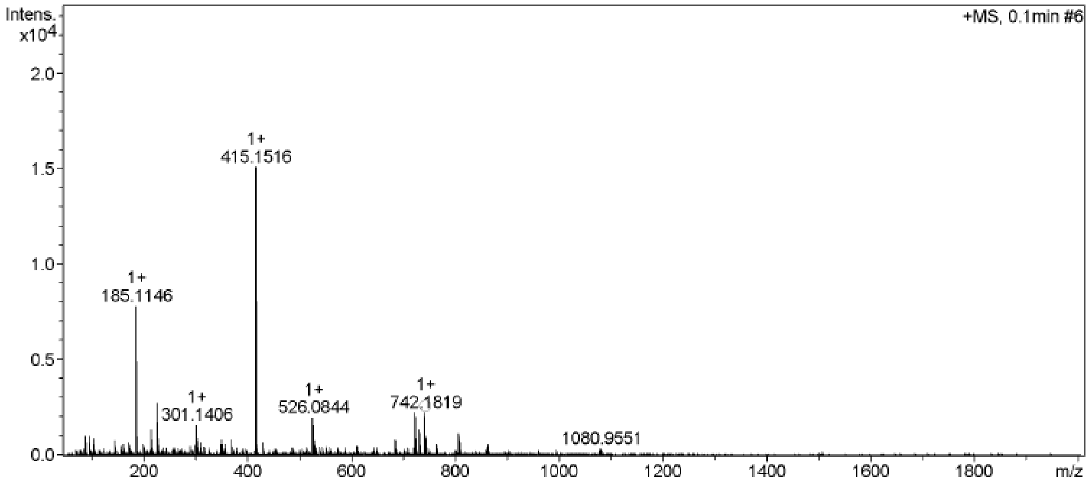


| Meas. m/z | # | Ion Formula | m/z | err [mDa] | err [ppm] | rdb | N-Rule | e⁻ Conf | mSigma |
|---|---|---|---|---|---|---|---|---|---|
| 742.181927 | 1 | C42H34BF2IN2 | 742.182952 | -1.0 | -1.4 | 26.0 | ok | odd | 15.6 |

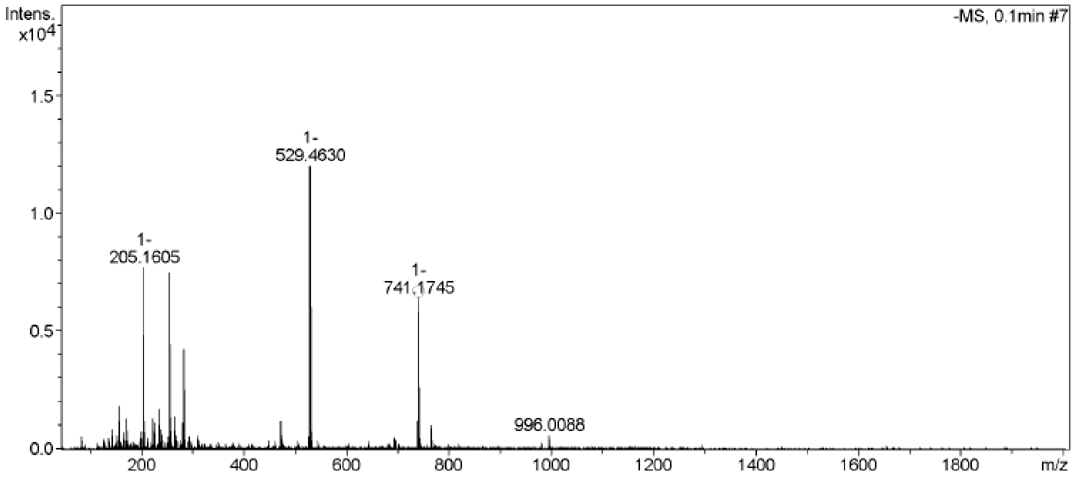


| Meas. m/z | # | Ion Formula | m/z | err [mDa] | err [ppm] | rdb | N-Rule | e⁻ Conf | mSigma |
|---|---|---|---|---|---|---|---|---|---|
| 741.174473 | 1 | C42H33BF2IN2 | 741.176224 | -1.8 | -2.4 | 26.5 | ok | even | 29.9 |

Figure S10. HR-MS (ESI-TOF) positive and negative scans of MB2PI.

## Crystallography

**Table S1 Crystal data and structure refinement for** $MB2P\cdot 0.475(CHCl_3)$

| | |
|---|---|
| Empirical formula | $C_{42.48}H_{35.48}BCl_{1.43}F_2N_2$ |
| Formula weight | 673.23 |
| Temperature/K | 100.01(11) |
| Crystal system | monoclinic |
| Space group | $P2_1/c$ |
| a/Å | 22.5919(8) |
| b/Å | 12.5812(5) |
| c/Å | 25.2321(9) |
| α/° | 90 |
| β/° | 96.352(3) |
| γ/° | 90 |
| Volume/$Å^3$ | 7127.8(5) |
| Z | 8 |
| $\rho_{calc}$ g/$cm^3$ | 1.255 |
| μ/$mm^{-1}$ | 1.584 |
| F(000) | 2812.0 |
| Crystal size/$mm^3$ | 0.609 × 0.043 × 0.032 |
| Radiation | Cu Kα (λ = 1.54184) |
| 2θ range for data collection/° | 7.05 to 133.202 |
| Index ranges | -26 ≤ h ≤ 26, -14 ≤ k ≤ 10, -28 ≤ l ≤ 30 |
| Reflections collected | 52467 |
| Independent reflections | 12557 [$R_{int}$ = 0.0886, $R_{sigma}$ = 0.0612] |
| Data/restraints/parameters | 12557/3043/1179 |
| Goodness-of-fit on $F^2$ | 1.059 |
| Final R indexes [I>=2σ (I)] | R1 = 0.1039, wR2 = 0.2897 |
| Final R indexes [all data] | R1 = 0.1305, wR2 = 0.3148 |
| Largest diff. peak/hole / e $Å^{-3}$ | 0.81/-0.40 |
| CCDC deposition number | 2157191 |

The data were collected at 100(1)K on a Synergy, Dualflex, AtlasS2 diffractometer using Cu*K*α radiation (λ = 1.54184 Å) and the *CrysAlis PRO* 1.171.40.67a suite[1]. Using SHELXLE[2] and Olex2[3] the structure was solved by dual space methods (SHELXT[4]) and refined on $F^2$ using all the reflections (SHELXL-2018/3[5]). All the non-hydrogen atoms were refined using anisotropic atomic displacement parameters, except for the carbon atoms of the overlapping, partial-occupancy chloroform solvate molecules and the 5% occupancy chlorine atoms.
SAME restraints were applied to all the perylene groups, to the disordered section of the BODIPY in mol 1, and to the chloroform molecules. Hydrogen atoms were inserted at calculated positions using a riding model. The crystals diffracted poorly and, despite a long collection time (*ca.* 80 hr), the data set is very weak and the structure shows significant disorder. Consequently, the precision of the structure determination is reduced but the main features are clear. Crystal parameters, data collection and structure refinement details are summarised in Table S1. CCDC 2157191 contains the supplementary crystallographic data for this paper. These data can be obtained free of charge from The Cambridge

Crystallographic Data Centre via www.ccdc.cam.ac.uk/data_request/cif.


1. Rigaku Oxford Diffraction, (2019), CrysAlisPro Software system, version 1.171.40.67a, Rigaku Corporation, Oxford, UK.
2. C.B. Hübschle, G.M. Sheldrick and B. Dittrich. J. Appl. Cryst., 2011, **44**, 1281-1284.
3. O.V. Dolomanov, L.J. Bourhis, R.J. Gildea, J.A.K. Howard & H. Puschmann. J. Appl. Cryst., 2009, **42**, 339-341
4. G.M. Sheldrick, Acta Cryst., 2015, **A71**, 3-8.
5. G.M. Sheldrick, Acta Cryst., 2015, **C71**, 3-8.


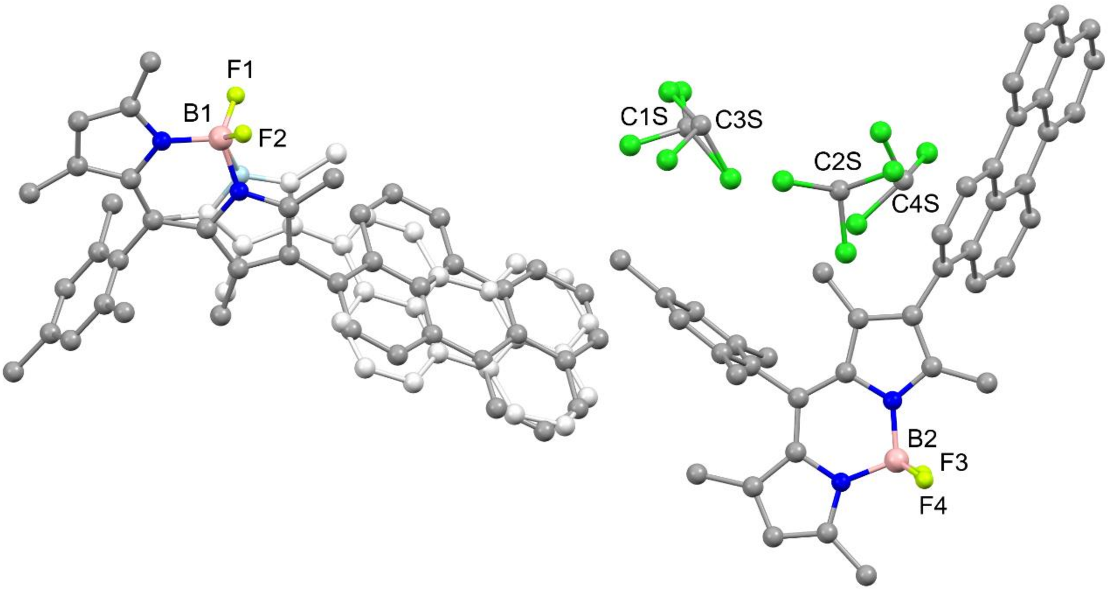


Figure S11. Asymmetric unit showing disorder in the molecule containing B1 (minor component shown in paler colours).

The asymmetric unit (above) contains two independent $C_{42}H_{35}BF_2N_2$ molecules and some chloroform solvate. In the molecule containing B1 (mol1), the perylene group is disordered by rotation of 180° about the C–C bond linking it to the BODIPY section and the site occupancies refined to 0.74 and 0.26 for the major and minor components, respectively. A slight displacement of the linked 5-membered ring allows a close spatial overlap of the two perylene components (Figures S12 & S13).

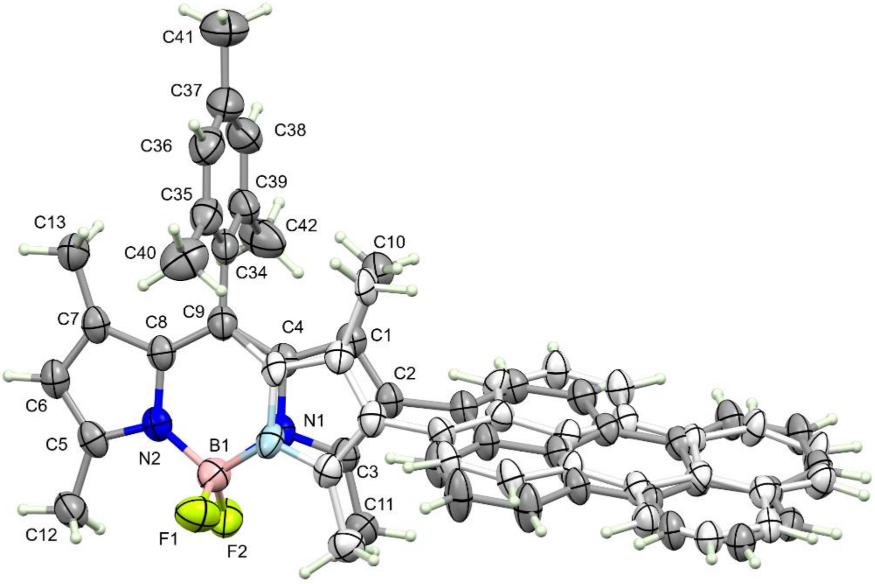


Figure S12. Disordered molecule 1 (50% probability ellipsoids) showing numbering scheme for the BODIPY section. Minor component shown in paler colours.

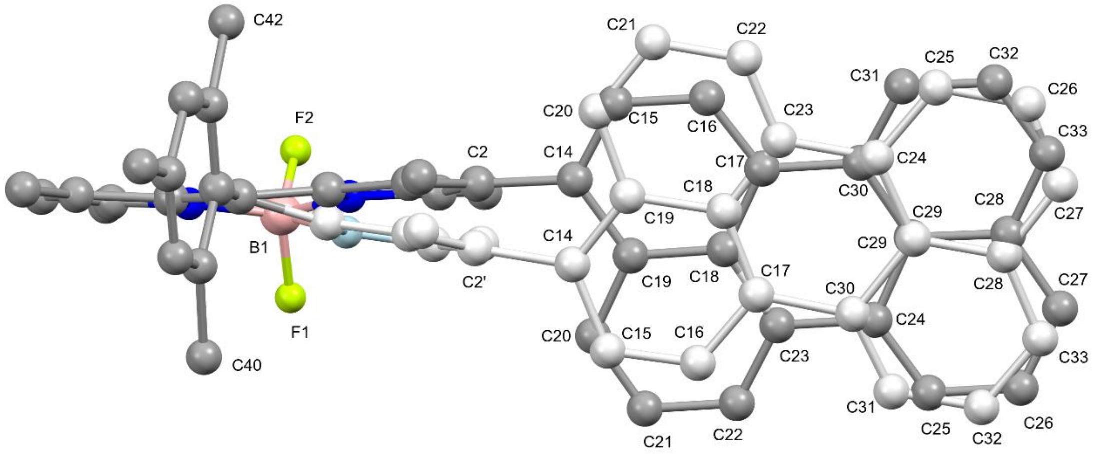


Figure S13. Disordered mol1 showing numbering scheme for the perylene sections. Minor component shown in paler colours.

The ADPs of the second molecule (mol2, containing B2) suggest a small amount of disorder in the perylene group (Figure S14) but this has not been modelled, given the low data quality and the more significant disorders elsewhere. The conformations of the two molecules are similar and the small differences (Figure S15) can probably be ascribed to intermolecular interactions in the solid state. In each case the mean plane of the perylene group is rotated with respect to the mean plane of the BODIPY core, the interplanar angles are 69.8(1)° and 73.5(3)° for the major and minor components of mol1 and 73.23(6)° for mol2.

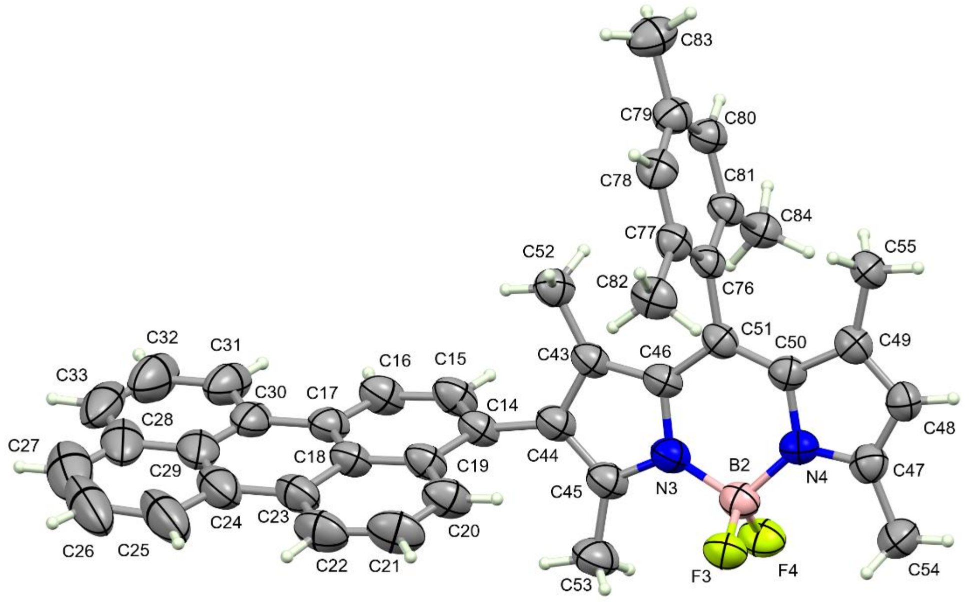


Figure S14. Mol 2 (50% probability ellipsoids) showing numbering scheme.

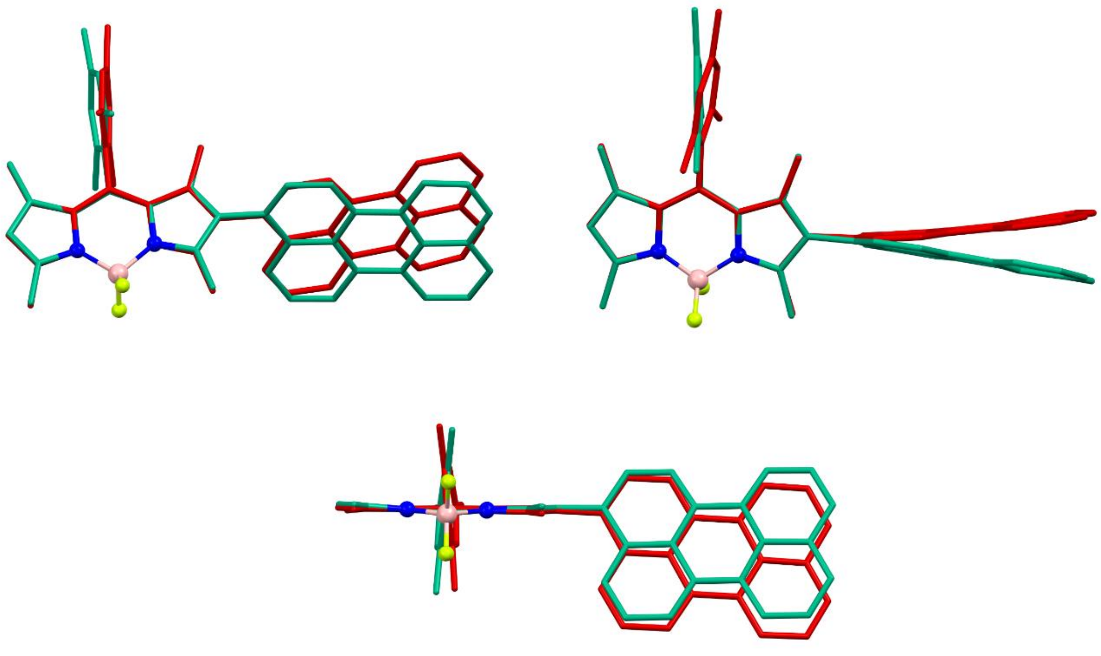

Figure S15. Overlays of mol1 (green) and mol2 (red), based on fitting the N and F atoms only. Only the major component of the disorder is shown for mol1 and it is inverted relative to the asymmetric unit coordinates (permitted in this centrosymmetric space group).

Mol1 is paired with a symmetry equivalent by inversion (symmetry operation 1-x, 1-y, 1-z) to generate π-stacked dimeric units; this stacking does not extend further through the structure (Figure S16). There are no equivalent π-interactions involving mol2 but there is a set of reasonably convincing ($sp^2$)C–H···F hydrogen bonds linking the mol2 units into chains running parallel to the *b* axis (Figure S17).

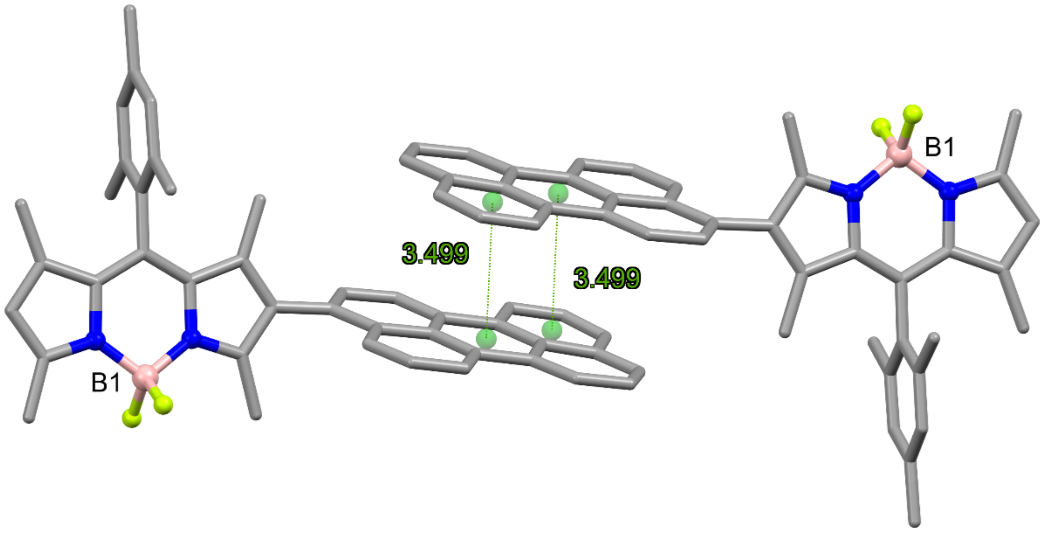


Figure S16. π-stacking showing centroid-centroid distances (Å) between mol1 and its symmetry equivalent under 1-x, 1-y, 1-z.

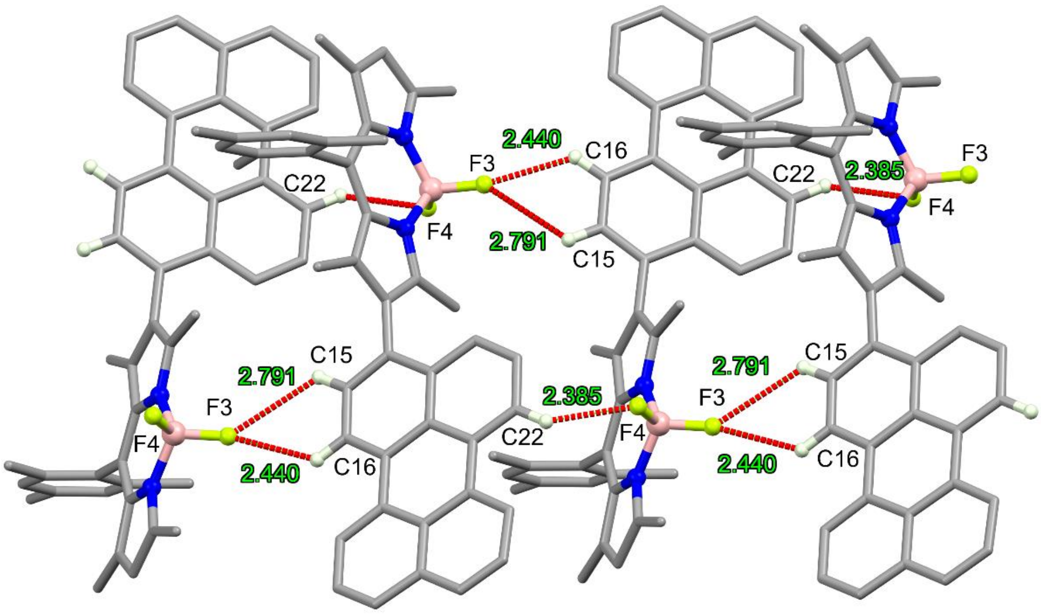


Figure S17. Dashed red lines indicate ($sp^2$)C–H···F interactions linking the mol2 units into chains running parallel to the *b* axis.

Examination of the unit cell packing (Figures S18 & S19) shows the solvate chloroform molecules lying in columns parallel to the b axis, the solvate was modelled over four overlapping sites with occupancies of 0.35, 0.40, 0.15 and 0.05 for the molecules cantered on C1S, C2S, C3S and C4S, respectively. The site occupancies were adjusted by hand to minimise the residual electron density in this region. Part-occupancy carbon atoms C1S, C2S and the 0.05 occupancy chlorine atoms were refined isotropically, while a fixed isotropic parameter was used for C3S and C4S.

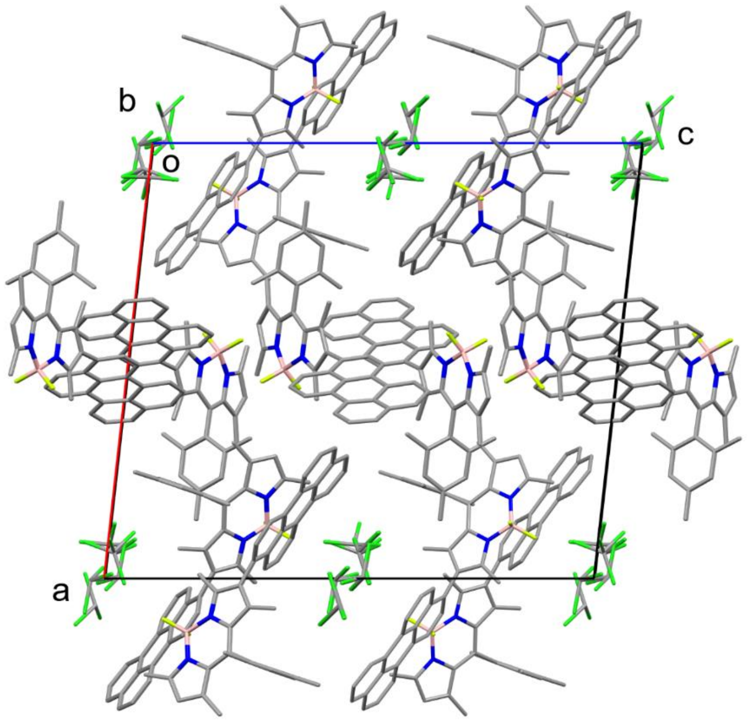


Figure S18. Unit cell packing viewed down the b axis. Perylene disorder and hydrogen atoms removed for clarity.

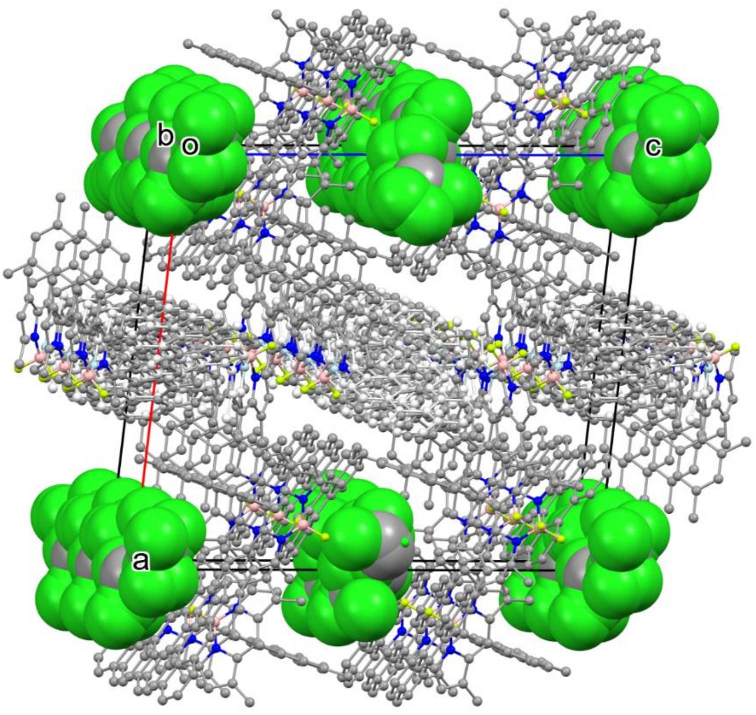


Figure S19. Unit cell packing showing partial-occupancy chloroform molecules (spacefill) in channels parallel to the b axis. Hydrogen atoms removed for clarity.

Photophysical Data

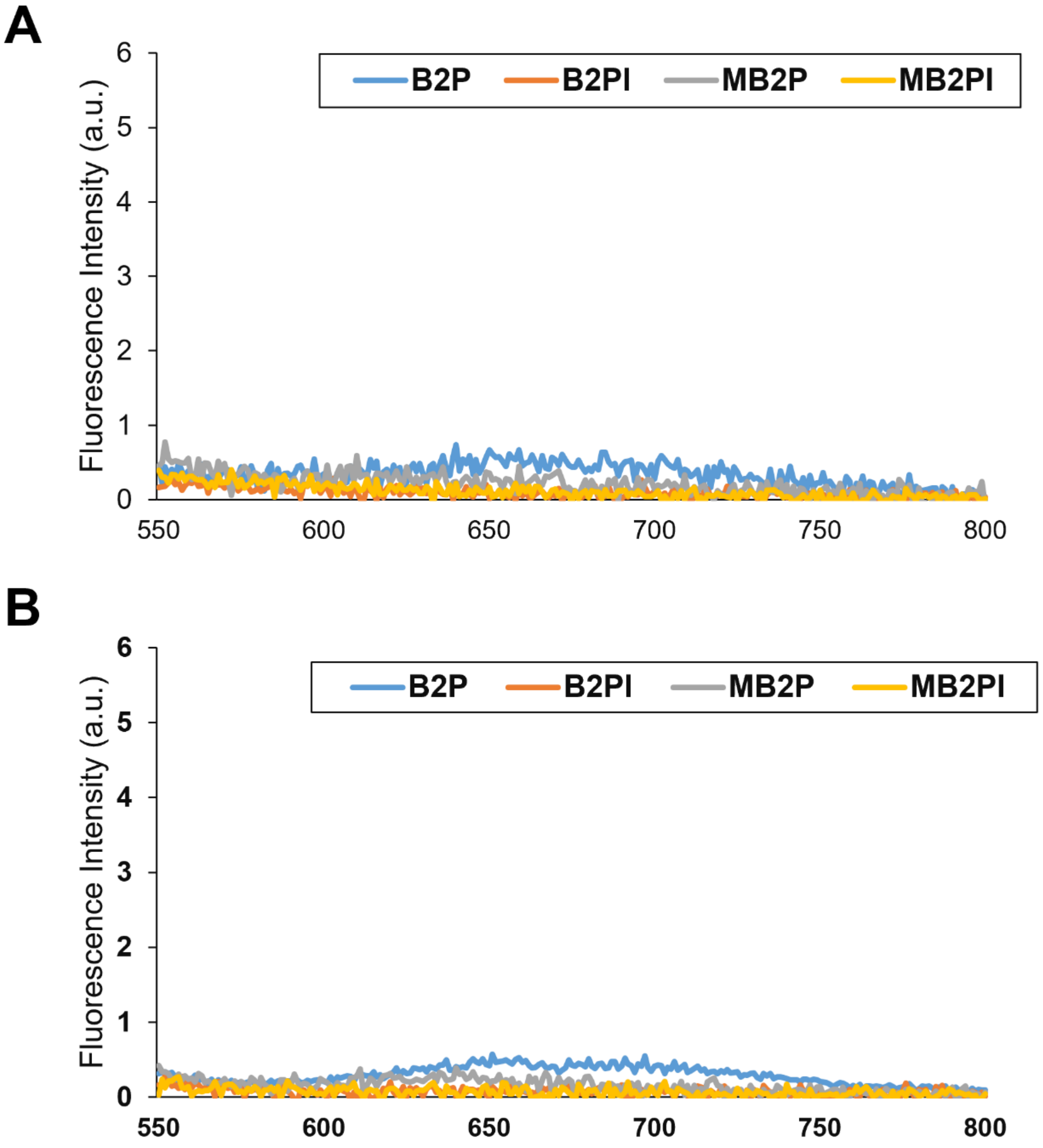


Figure S20. Emission of B2P, B2PI, MB2P and MB2PI (10 μM) in (A) phenol red free DMEM cell culture media and (B) PBS with 10% DMSO. B2P/MB2P were excited at 515 nm and B2PI/MB2PI excited at 532 nm and data was recorded with a slit width of 2.5-2.5 nm to match the photophysical studies reported in Figures, 1, 2 and Table 1.

## Response to Viscosity in Solution

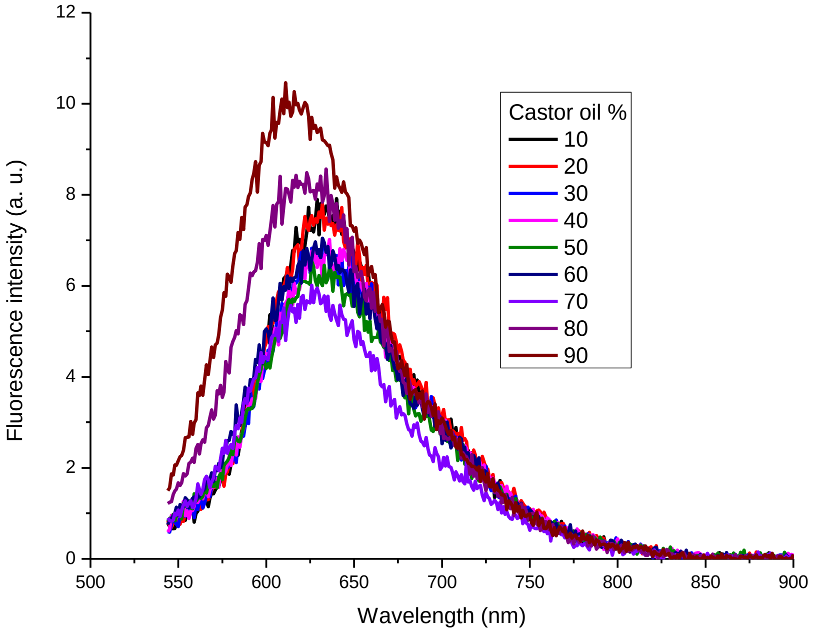


Figure S21. Emission of MB2PI (10 μM) in toluene with varying concentrations of castor oil

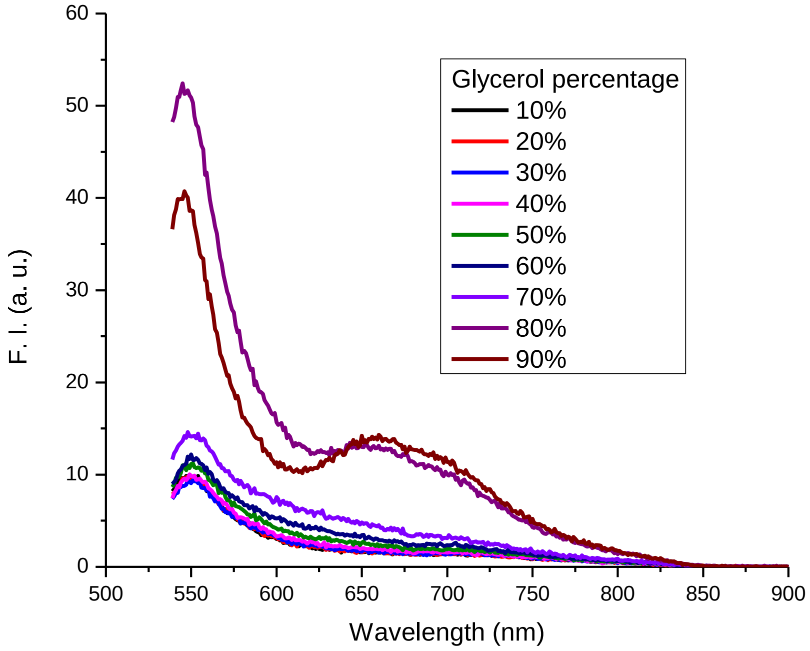


Figure S22. Emission of MB2PI (10 μM) in methanol with varying concentrations of glycerol where F.I. indicates fluorescence intensity.

## Uptake in Live Cell Monolayers

CHO cells were incubated in 75cm$^2$ cell culture flasks (Corning) with DMEM/F-12 Ham medium (Sigma-Aldrich) with L-glutamine and sodium bicarbonate supplemented with 10% fetal bovine serum (FBS) (Gibco) and 1% penicillin-streptomycin (Sigma-Aldrich). MCF-7 cells were incubated in 25cm$^2$ cell culture flasks with RPMI-1640 medium (Sigma-Aldrich) with L-glutamine and sodium bicarbonate supplemented with 10% fetal bovine serum (FBS, Gibco) and 1% penicillin-streptomycin. Cells were incubated at 37 °C with 5% $CO_2$ and detached from the flask at 90% confluency using a 1X Trypsin-EDTA solution (Sigma-Aldrich). Cells were counted using the EVE automated cell counter (NanoEnTek) and seeded at 1 x 10$^5$ cells per mL in a 4 or 8 chamber µ-slide (ibidi) for cell imaging and left for 24 h at 37 °C. Cells were rinsed with 1X PBS (Gibco) and stained with B2P or B2PI at 10 µM and MB2P or MB2PI at 30 µM for 17 h. Cells were washed twice with PBS and covered with phenol red free RPMI medium (Gibco) for imaging. Live cells were imaged using a Leica SP8 confocal microscope with 63x oil immersion lens and heated stage at 37 °C.

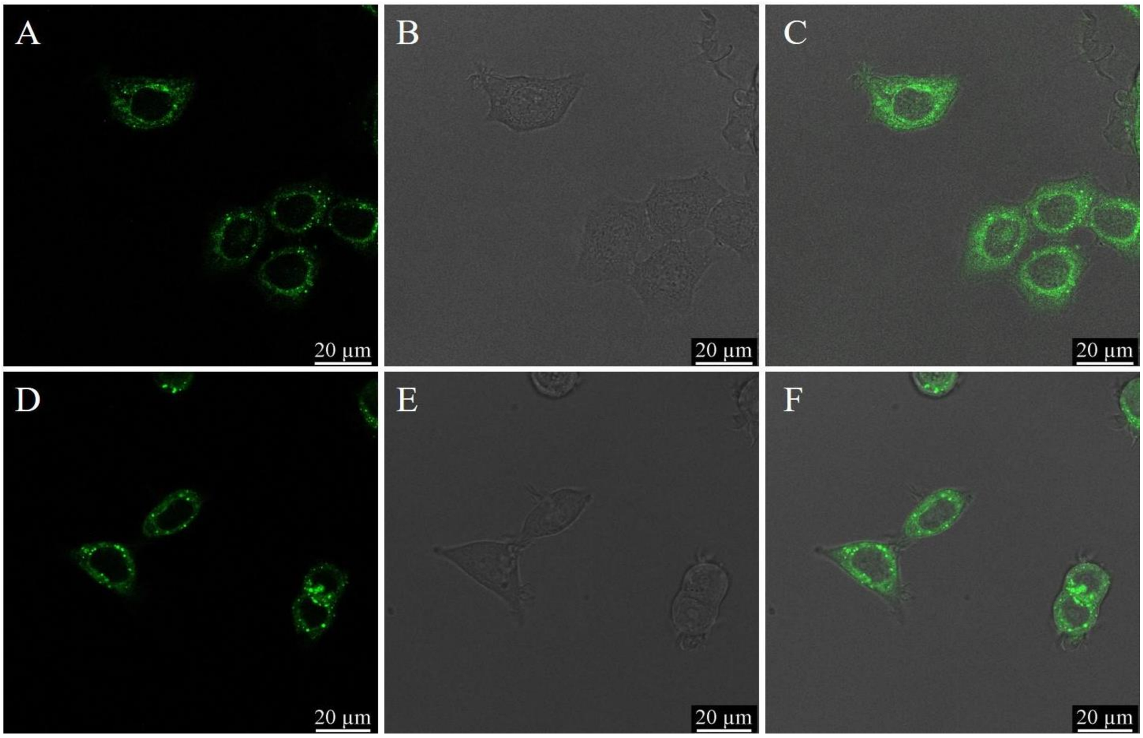


Figure S23. Confocal imaging of 10 µM B2P in MCF-7 cells after (A-C) 4 and (D-F) 17 h incubation.

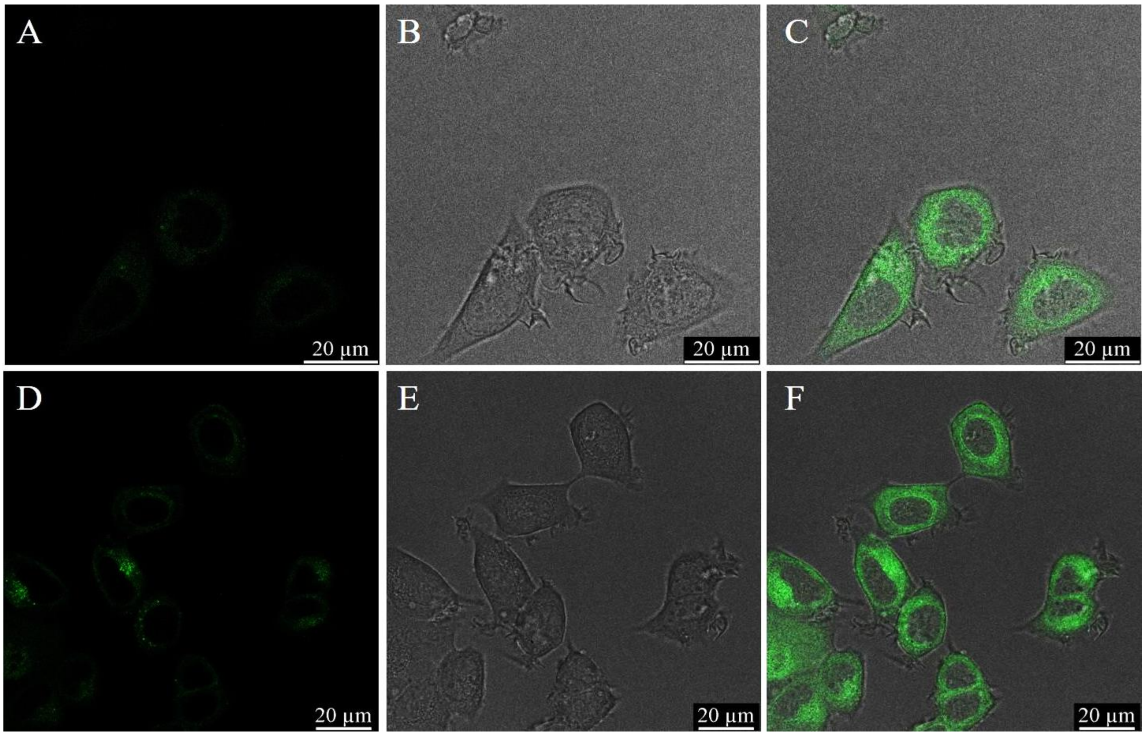


Figure S24. Confocal imaging of 10 µM B2PI in MCF-7 cells after (A-C) 4 and (D-F) 17 h incubation.

## Localisation in Live Cell Monolayers

For co-localisation studies, LysoTracker Deep Red, ER Tracker Blue-White, PyLa-C17Cer or BODIPY 493/503 were added to CHO and MCF-7 cells after the 17 h incubation period with the BODIPY probes. Cells were washed twice with PBS and imaged in phenol red free media. The BODIPY-perylene compounds were excited at their respective $\lambda_{abs}$ and emission for co-localisation studies was collected from 580-640 nm, where the emission collection window was shortened depending on the $\lambda_{em}$ of the BODIPY derivative being tested to ensure minimal overlap with the commercial dyes. Moreover, images were obtained by sequential sequencing with gating applied. The conditions for co-localisation studies are as follows: LysoTracker Deep Red (100 nM, 1 h) Ex 647, Em 665-685 nm; ER Tracker Blue/White (1 µM, 45 minutes) Ex 405 nm, Em 435-560 nm; PyLa-C17Cer (2 µM, 2 h) Ex 405 nm, Em 430-550 nm and BODIPY 493/503 (2 µM, 30 min) Ex 493 nm, Em 500-515 nm. Co-localisation analysis was performed using ImageJ.

| BODIPY | Cell Line | Pearson Coefficient (r) | | | |
|---|---|---|---|---|---|
| | | LysoTracker Deep Red | ER Tracker Blue/White | PyLa-C17Cer | BODIPY 493/503 |
| **B2P** | CHO<br>MCF-7 | 0.34 ± 0.04<br>0.75 ± 0.11 | 0.17 ± 0.06<br>0.14 ± 0.07 | 0.69 ± 0.03<br>0.55 ± 0.07 | 0.78 ± 0.09<br>0.75 ± 0.07 |
| **B2PI** | CHO<br>MCF-7 | 0.65 ± 0.05<br>0.71 ± 0.03 | 0.46 ± 0.04<br>0.19 ± 0.05 | 0.65 ± 0.04<br>0.54 ± 0.05 | |
| **MB2P** | CHO<br>MCF-7 | 0.59 ± 0.08<br>0.57 ± 0.06 | 0.56 ± 0.02<br>0.54 ± 0.04 | 0.67 ± 0.09<br>0.67 ± 0.03 | 0.70 ± 0.03<br>0.66 ± 0.11 |
| **MB2PI** | CHO<br>MCF-7 | 0.44 ± 0.07<br>0.59 ± 0.09 | 0.46 ± 0.01<br>0.53 ± 0.04 | 0.33 ± 0.06<br>0.49 ± 0.04 | 0.23 ± 0.04<br>0.45 ± 0.06 |

Table S2. Pearson's coefficient values of the BODIPY-perylene compounds in CHO and MCF-7 cells with LysoTracker Deep Red (100 nM, 1 h), ER Tracker Blue/White (1 µM, 45 min), PyLa-C17Cer (2 µM, 2 hours) and BODIPY 493/503 (2 µM, 30 min)

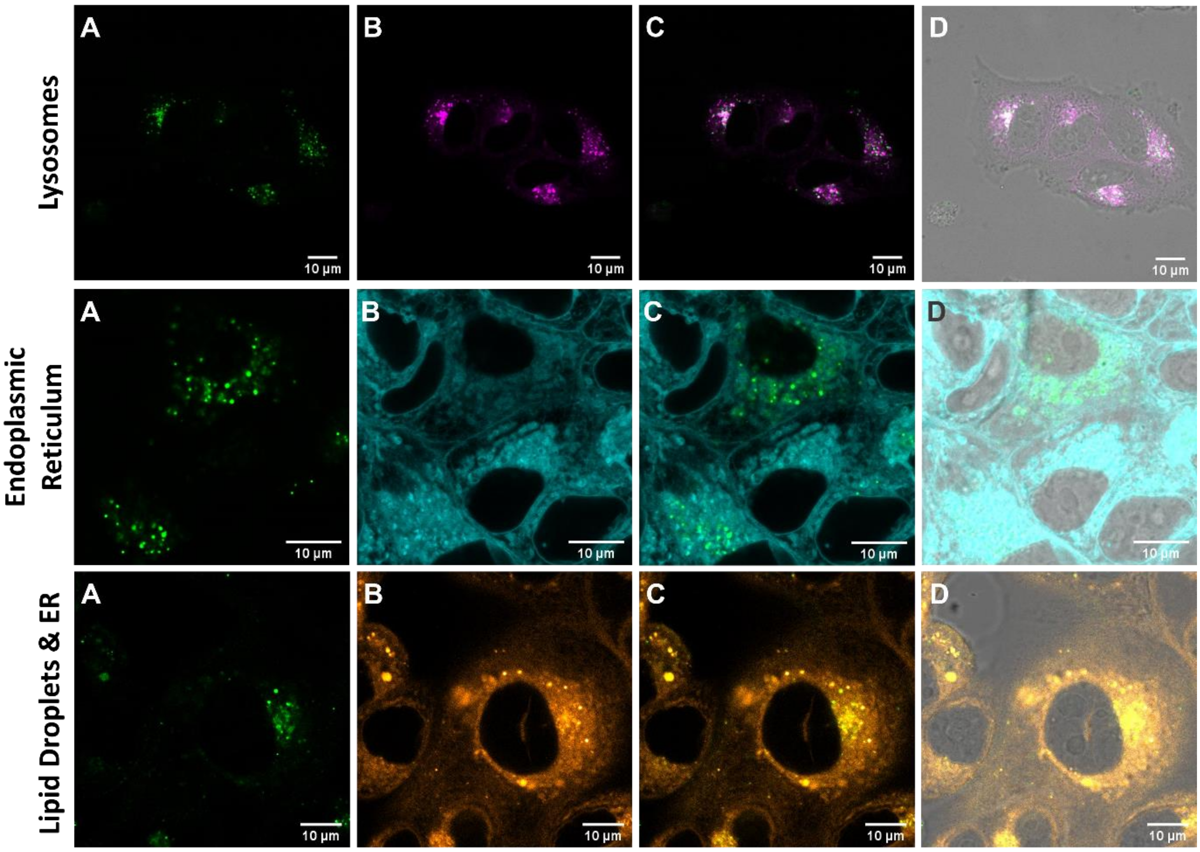


Figure S25. Co-localisation imaging of B2PI (10 µM, 17 h) with LysoTracker Deep Red, ER Tracker Blue/White and PyLa-C17Cer. Images are labelled with the corresponding dyes in MCF-7 cells, where (A) is the B2PI channel, (B) indicates the co-localisation dyes where LysoTracker is indicated by magenta, ER Tracker Blue/White is cyan and PyLa-C17Cer is orange. The overlay is shown with and without the phase contrast channel in (C) and (D).

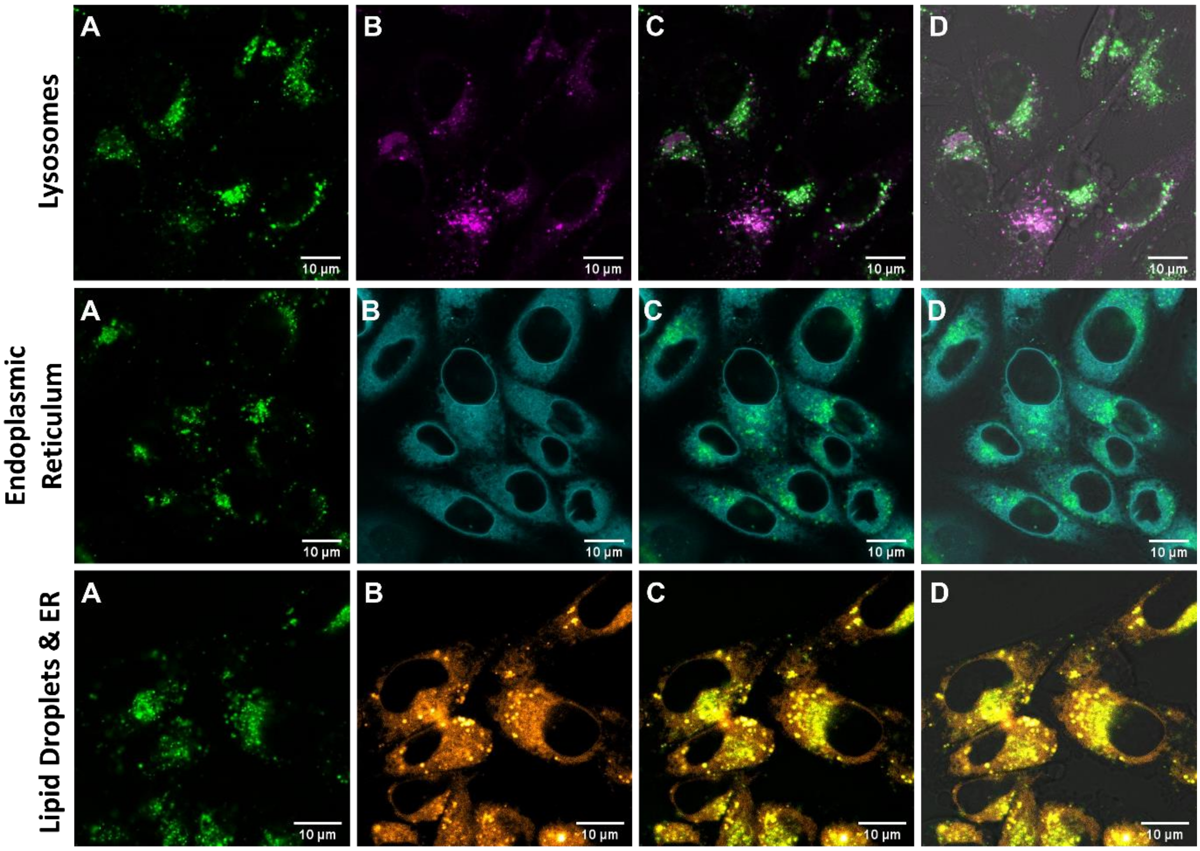


Figure S26. Co-localisation imaging of B2PI (10 µM, 17 h) with LysoTracker Deep Red, ER Tracker Blue/White and PyLa-C17Cer. Images are labelled with the corresponding dyes in CHO cells, where (A) is the B2PI channel, (B) indicates the co-localisation dyes where LysoTracker is indicated by magenta, ER Tracker Blue/White is cyan and PyLa-C17Cer is orange. The overlay is shown with and without the phase contrast channel in (C) and (D).

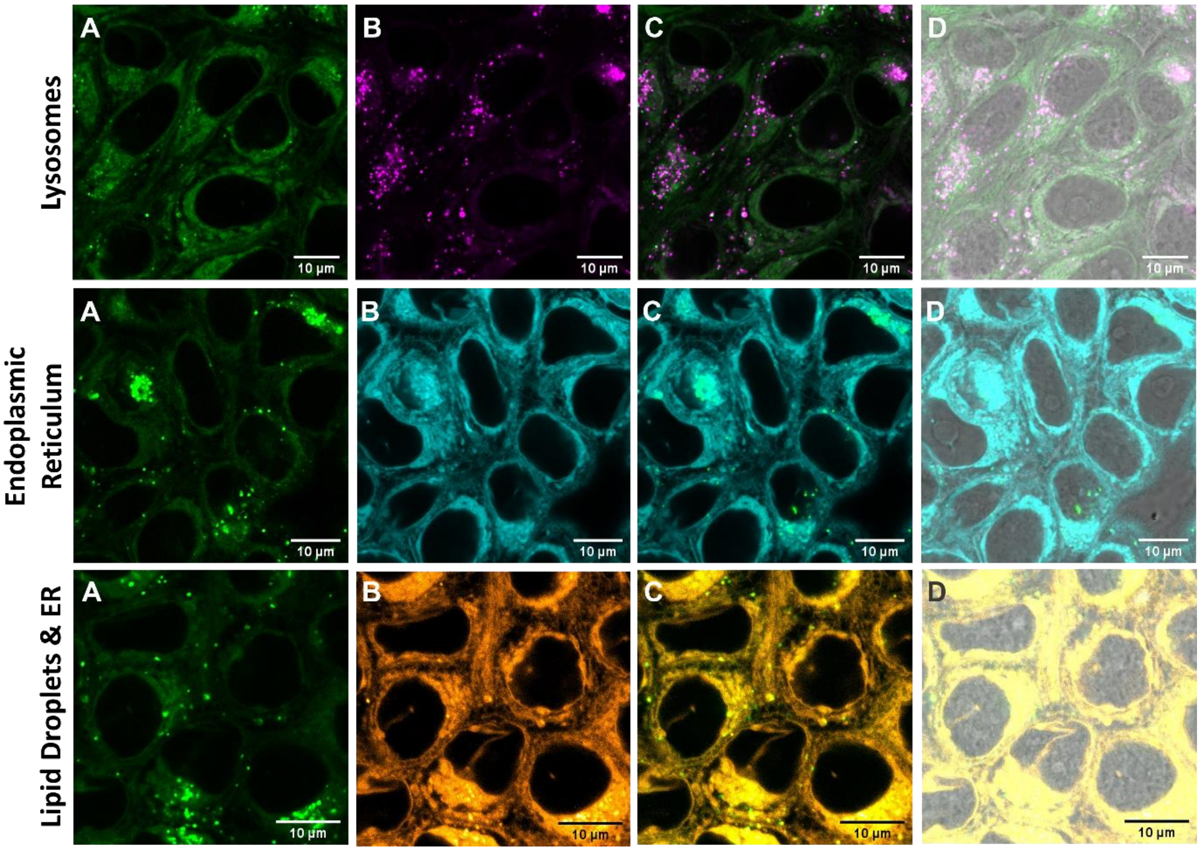


Figure S27. Co-localisation imaging of M2P (30 µM, 17 h) with LysoTracker Deep Red, ER Tracker Blue/White and PyLa-C17Cer. Images are labelled with the corresponding dyes in MCF-7 cells, where (A) is the B2PI channel, (B) indicates the co-localisation dyes where LysoTracker is indicated by magenta, ER Tracker Blue/White is cyan and PyLa-C17Cer is orange. The overlay is shown with and without the phase contrast channel in (C) and (D).

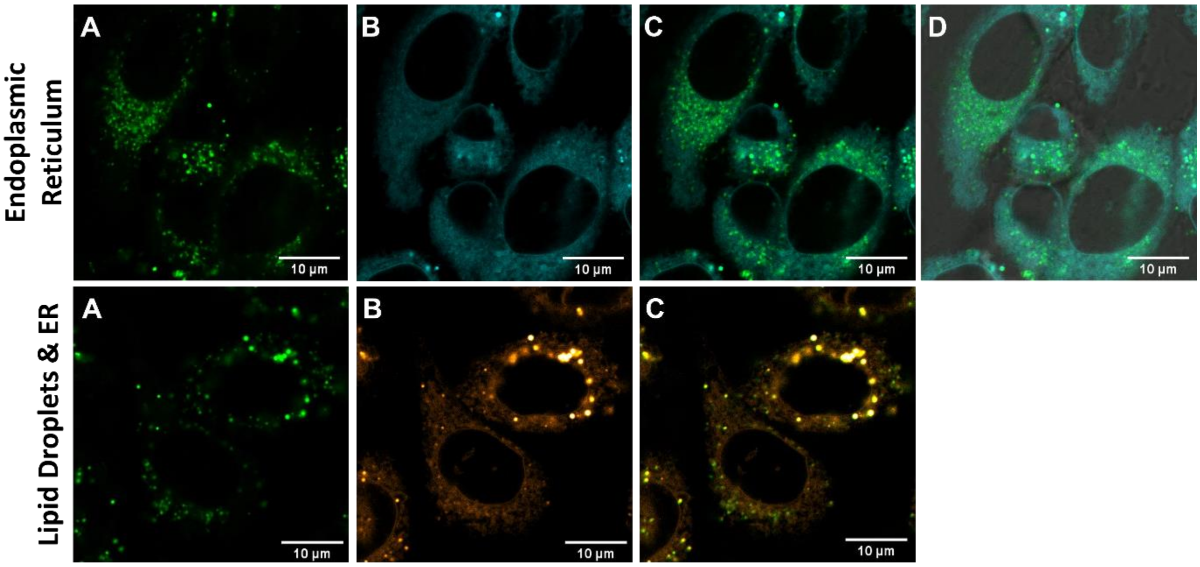


Figure S28. Co-localisation imaging of M2P (30 µM, 17 h) with ER Tracker Blue/White and PyLa-C17Cer. Images are labelled with the corresponding dyes in CHO cells, where (A) is the B2PI channel, (B) indicates the co-localisation dyes where LysoTracker is indicated by magenta, ER Tracker Blue/White is cyan and PyLa-C17Cer is orange. The overlay is shown with and without the phase contrast channel in (C) and (D).

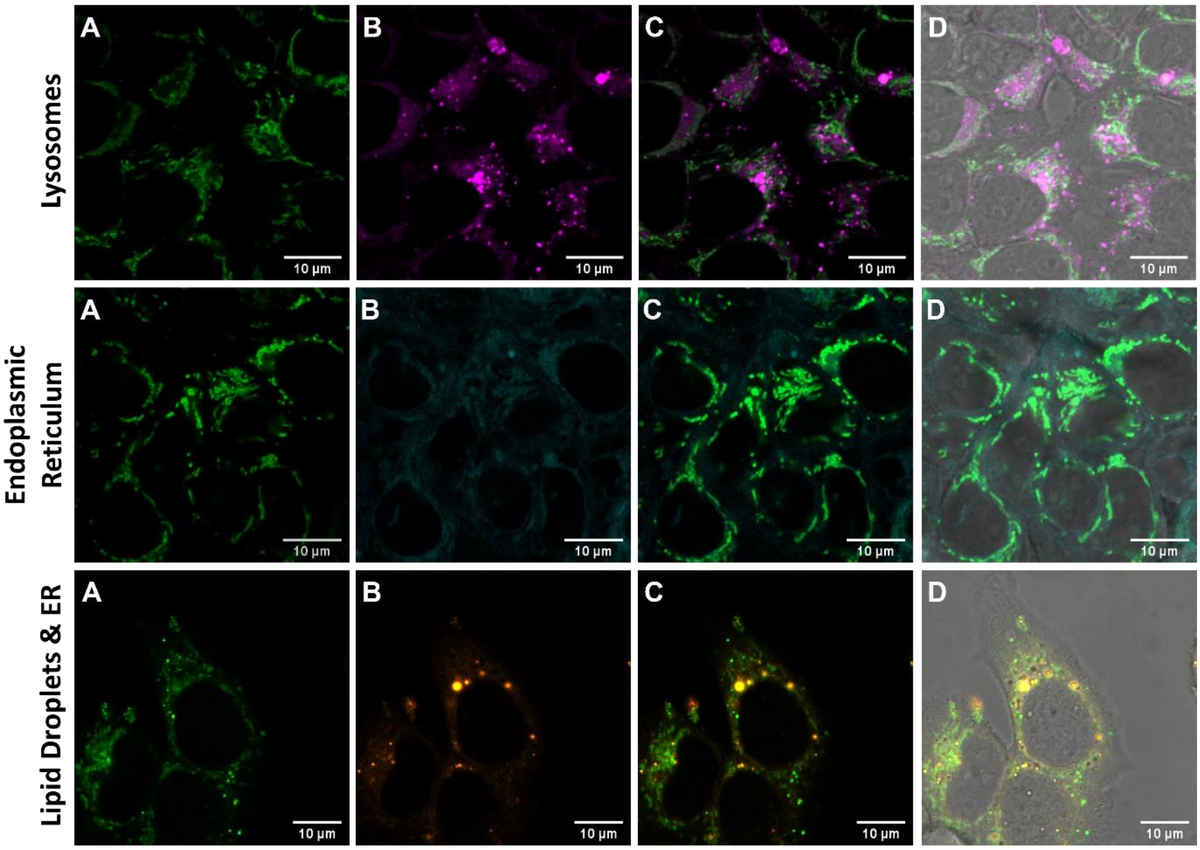


Figure S29. Co-localisation imaging of MB2PI (30 µM, 17 h) with LysoTracker Deep Red, ER Tracker Blue/White and PyLa-C17Cer. Images are labelled with the corresponding dyes in MCF-7 cells, where (A) is the B2PI channel, (B) indicates the co-localisation dyes where LysoTracker is indicated by magenta, ER Tracker Blue/White is cyan and PyLa-C17Cer is orange. The overlay is shown with and without the phase contrast channel in (C) and (D).

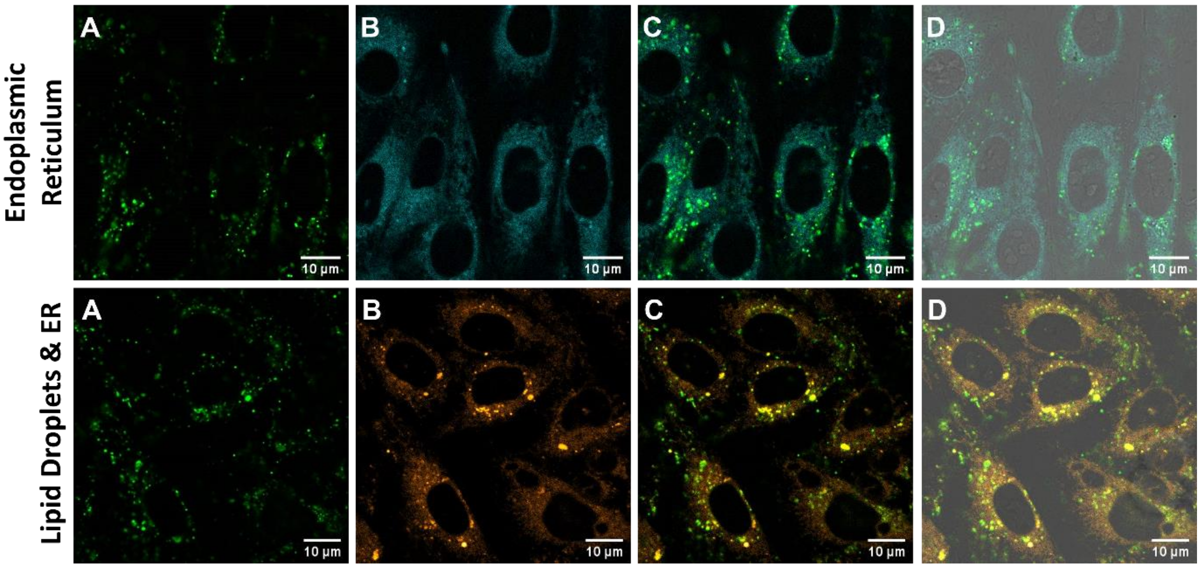


Figure S30. Co-localisation imaging of MB2PI (30 µM, 17 h) with ER Tracker Blue/White and PyLa-C17Cer. Images are labelled with the corresponding dyes in CHO cells, where (A) is the B2PI channel, (B) indicates the co-localisation dyes where ER Tracker Blue/White is cyan and PyLa-C17Cer is orange. The overlay is shown with and without the phase contrast channel in (C) and (D).

## Cellular Response to TNF-α

Chinese Hamster Ovary (CHO) non-cancer and MCF-7 breast cancer cells were seeded in black bottomed cell culture treated 96 well plates (Nunc) at 1 x $10^4$ cells per 100 µL and left for to grow and attach for 24 h at 37°C and 5 % $CO_2$. The cells were treated with TNF-α at 100 ng/mL in cell culture media for 0-24 hours prior to the addition of MB2PI (10 µM, 17 h). After the 17 h incubation with MB2PI, the cells were washed twice with 1X PBS (pre-warmed to 37°C) and 100 µL PBS added to the wells. A control with untreated cells in PBS was added to the plates for background subtraction. The emission spectra were measured using a BMG LABTECH CLARIOstar that was set to 37°C.

## Cytotoxicity Studies

Chinese Hamster Ovary (CHO) non-cancer and MCF-7 breast cancer cells were seeded in clear cell culture treated 96 well plates (Nunc) at 1 x $10^4$ cells per 100 µL and left for to grow and attach for 24 h at 37°C and 5 % $CO_2$. For 24 h dark toxicity studies, 50, 10, 5 and 1 µM stock concentrations of B2P, B2PI, MB2P and MB2PI were prepared in media to a final DMSO concentration of ≤1%. 100 µL of each concentration was added to the wells in triplicate and the plates incubated for 24 h in the dark at 37°C. Cell viability of all plate-based studies was assessed using the alamar blue assay. A 10% Resazurin-media solution (alamarBlue, Invitrogen) was added to the wells and incubated for 5 h in the absence of light. Absorbance was measured using a BMG LABTECH CLARIOstar plate reader at 570 nm and 600 nm (corrected for background subtraction). Cell viability is presented as a percentage (%) compared to untreated control wells. Plates were performed in triplicate.

## Phototoxicity Studies

Phototoxicity and dark control plates were performed by incubating CHO or MCF-7 cells with B2P, B2PI, MB2P and MB2PI for 17 h to allow uptake, emptying the wells, replacing with phenol red free media, and irradiating at a total dose of 17 ± 1.64 J/$cm^2$ (2 h at 2.37 ± 0.23 mW/$cm^2$) using a 470 nm LED (TeleOpto LEDA-X LED driver and array). Control plates were incubated in the dark during the irradiation period (2 h). After irradiation, the cells were incubated in the dark overnight to allow for cell recovery. Irradiation conditions were tested on untreated cells to confirm cells remained viable at this dose before any experiments were performed and each phototoxicity plate included untreated test wells for cell viability comparison. Viability was determined using the alamar blue assay as per the cytotoxicity protocol.

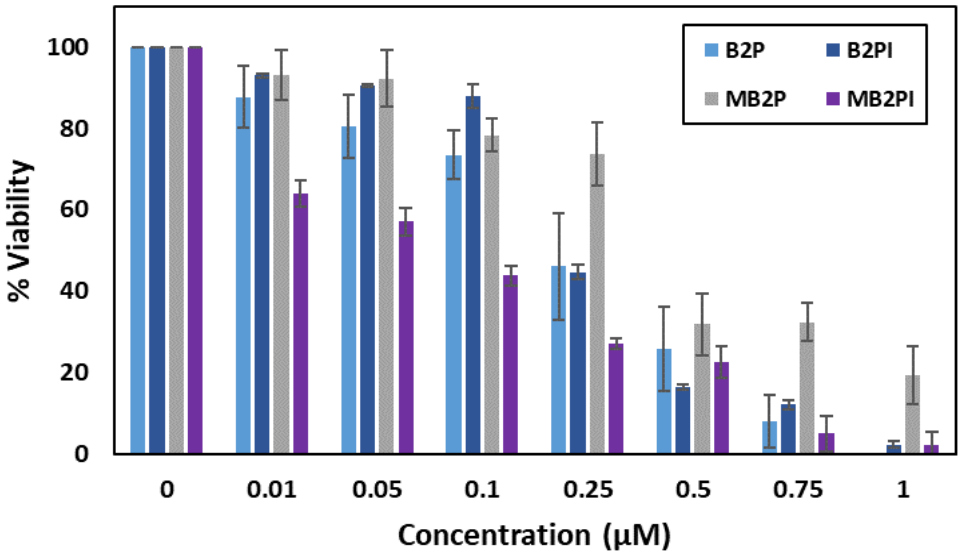


Figure S31. Phototoxicity results of MCF-7 cells. Cells were irradiated using a 470 nm LED and cell viability determined using the alamar blue assay. % Viability was determined relative to untreated control cells.

## DPBF Singlet $O_2$ study

To assess singlet oxygen ($^1O_2$) production and determine if the phototoxicity of our BODIPY complexes was caused by the generation of reactive oxygen species (ROS), a $^1O_2$ scavenger study was completed using 1,3-diphenylisobenzofuran (DPBF). A 20 mM solution of DPBF in methanol was prepared, and stocks of the BODIPY-perylene compounds in DMSO were prepared under absorbance matched concentrations, starting at a 30 μM concentration of each. Test solutions of 1:1, DPBF: BODIPY in methanol (final concentration of 10 mM DPBF) were added to a clear 96 well plate (Nunc) and absorbance was measured using a BMG LABTECH CLARIOstar plate reader. Each test condition was performed in triplicate.

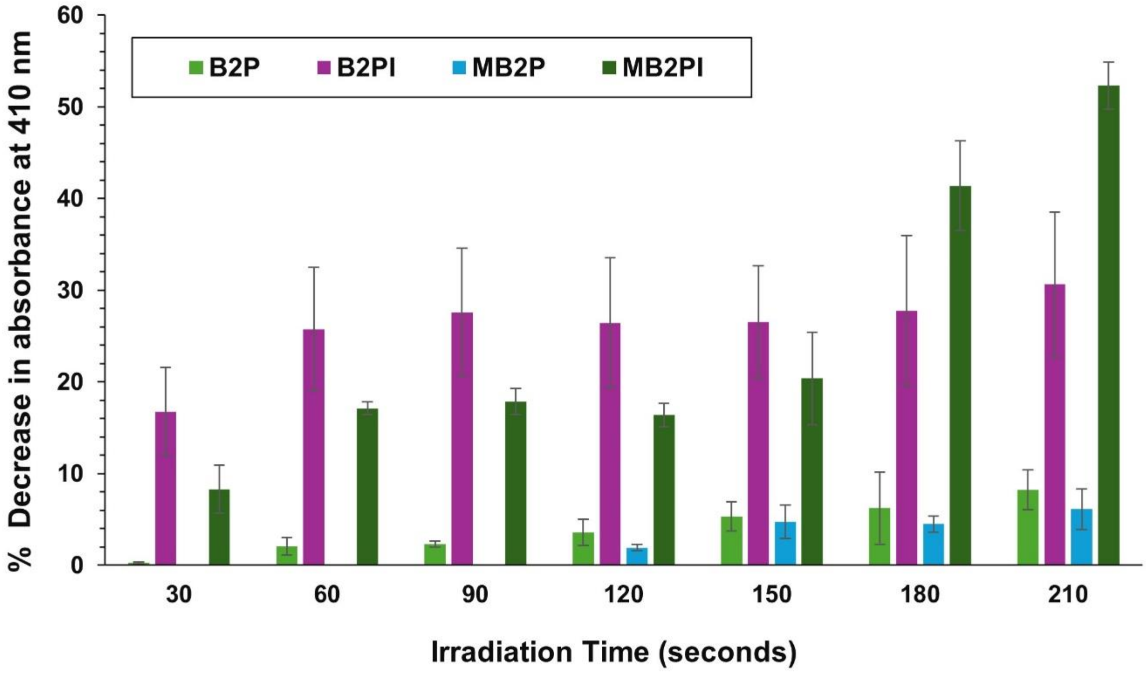


Figure S32. DPBF (10 mM) $^1O_2$ scavenger assay with B2P, MB2P, B2PI or MB2PI in methanol from 0-210 seconds irradiation, where absorbance at 410 nm was recorded every 30 seconds.

## ROS Imaging Studies

CHO and MCF-7 cells were treated with MB2PI (30 μM, 17 h) for the ROS imaging studies. A stock of 5 mM $H_2$DCFDA was prepared in DMSO and diluted to 5 μM in PBS. The cells were treated with the 5 μM $H_2$DCFDA-PBS solution for 30 minutes at 37 $^oC$ and 5% $CO_2$ prior to imaging. The cells were then washed twice with PBS and imaged in phenol red free media (F12-Ham/DMEM for CHO cells and RPMI for MCF-7) supplemented with 10% FBS.

A 63x oil immersion objective was used to acquire images. The irradiance was determined using a power meter from Edmund Optics.

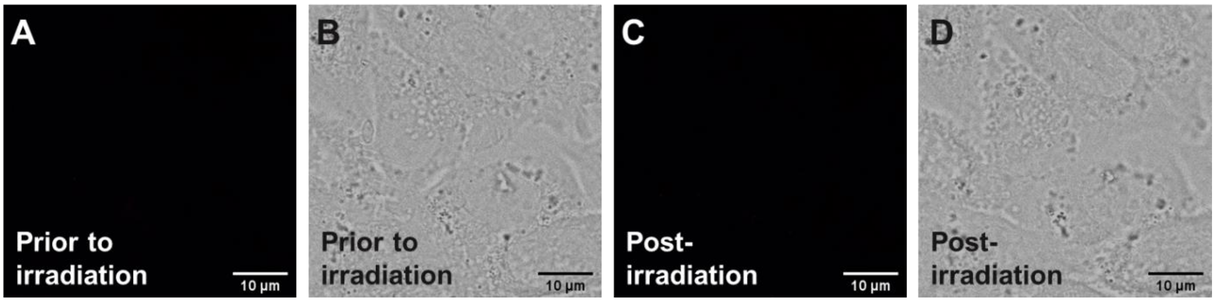


Figure S33. Confocal imaging of CHO cells (A-B) prior and (C-D) post irradiation confirming untreated control cells do not produce ROS under the irradiation conditions used for ROS studies with MB2PI. A and C depict the $H_2$DCFDA channel while B and D reflect the corresponding phase contrast image of the cells being imaged. 63x oil.

## Hypoxic Phototoxicity Studies

Hypoxic phototoxicity studies were performed as per routing phototoxicity protocol. Hypoxia was chemically induced using cobalt chloride ($CoCl_2$). A 1 M stock of $CoCl_2$ in sterile 1X PBS was prepared and further diluted to give a 1 mM working stock. The 1 mM stock was used to make up a 300 µM solution of $CoCl_2$ in media to induce a hypoxic environment in the cells. $CoCl_2$ was prepared fresh for each replicate. Cells were treated with $CoCl_2$ (300 µM) for 4 h prior to irradiation to ensure a hypoxic environment.

## 3D Cell Studies: Imaging

MCF-7 cells were seeded in a spheroid perfusion µ-slide (ibidi) at $2x10^5$ cells/mL and allowed to grow and compact for 72 h before being treated with 50 µM B2P or MB2PI for 17 h. The BODIPY-media solution was replaced with fresh phenol red free media prior to imaging. Spheroid images were captured at different planes in the z direction from below to above the spheroid and were acquired with a 40x oil immersion objective.

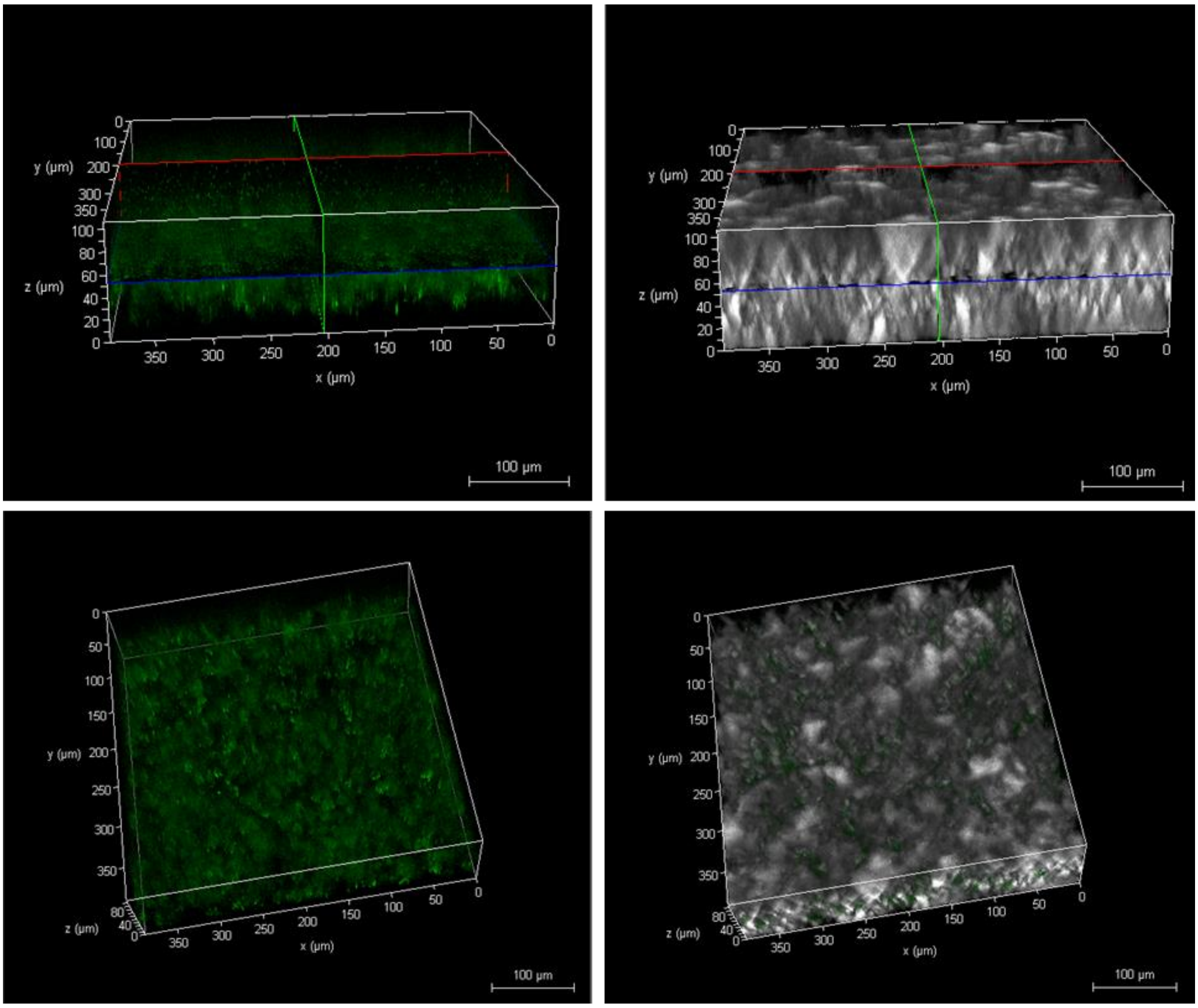


Figure S34. 3D rendering of B2P in MCF-7 spheroid showing deep penetration throughout the entire spheroid. 40x oil objective.

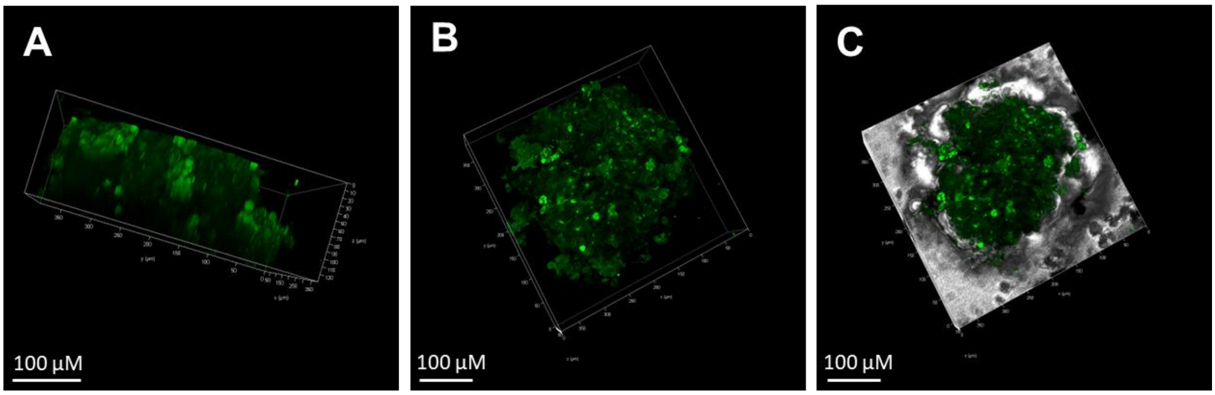


Figure S35. 3D rendering of B2PI (50 μM, 17 h) in MCF-7 spheroids showing deep penetration as depicted in (A) and (B) where (C) is the overlay of (B) with the corresponding phase contrast channel. 40x oil.

## 3D Cell Studies: Toxicity Assays

Spheroids were seeded in a clear BIOFLOAT™ round bottom 96 well plate (Sarstedt) at $2x10^4$ cells per well, centrifuged using the BMG Labtech CLARIOstar platereader for 5 minutes at 700 rpm and allowed to grow and compact for 96 hours. A 2X concentrated solution of B2P, B2PI, MB2P or MB2PI was added in a 1:1 ratio (BODIPY:media) to each well of the 96 well plate, which contained 50 µL phenol red free media, and incubated for 24 h. Prior to irradiation 90% of the media/Ru solution was removed, replaced with fresh phenol red media to dilute the remaining BODIPY (multiple dilution steps, more required for 15 µM and above), and brought to a total volume of 100 µL. Phototoxicity studies in HeLa spheroids were performed as per the cell monolayer protocol with an irradiation dose of 5 $J/cm^2$ using a 470 nm LED. Control studies with untreated spheroids confirmed 17 $J/cm^2$ irradiation dose was toxic to spheroids, however the spheroids remained viable after 5 $J/cm^2$ irradiation. CellTiter-Glo 3D cell viability assay (Promega) was used to assess spheroid viability and performed as per the manufacturer's protocol, where the CellTiter-Glo 3D reagent was added to the spheroid wells in a 1:1 ratio.
